# Associations between reproductive history, hormone use, *APOE* ε4 genotype and cognition in middle- to older-aged women from the UK Biobank

**DOI:** 10.3389/fnagi.2022.1014605

**Published:** 2023-01-19

**Authors:** Linn R. S. Lindseth, Ann-Marie G. de Lange, Dennis van der Meer, Ingrid Agartz, Lars T. Westlye, Christian K. Tamnes, Claudia Barth

**Affiliations:** ^1^NORMENT, Institute of Clinical Medicine, University of Oslo, Oslo, Norway; ^2^LREN, Department of Clinical Neurosciences, Centre for Research in Neurosciences, Lausanne University Hospital (CHUV) and University of Lausanne, Lausanne, Switzerland; ^3^Department of Psychology, University of Oslo, Oslo, Norway; ^4^Department of Psychiatry, University of Oxford, Oxford, United Kingdom; ^5^School of Mental Health and Neuroscience, Faculty of Health, Medicine and Life Sciences, Maastricht University, Maastricht, Netherlands; ^6^Department of Psychiatric Research, Diakonhjemmet Hospital, Oslo, Norway; ^7^Department of Clinical Neuroscience, Centre for Psychiatry Research, Stockholm Health Care Services, Karolinska Institute, Stockholm County Council, Stockholm, Sweden; ^8^NORMENT, Division of Mental Health and Addiction, Oslo University Hospital, Institute of Clinical Medicine, University of Oslo, Oslo, Norway; ^9^Department of Psychology, PROMENTA Research Center, University of Oslo, Oslo, Norway

**Keywords:** women’s health, cognition, population-based, pregnancy, hormonal contraceptives, hormone therapy, big data

## Abstract

**Introduction:**

Relative to men, women are at a higher risk of developing age-related neurocognitive disorders including Alzheimer’s disease. While women’s health has historically been understudied, emerging evidence suggests that reproductive life events such as pregnancy and hormone use may influence women’s cognition later in life.

**Methods:**

We investigated the associations between reproductive history, exogenous hormone use, apolipoprotein (*APOE*) ε4 genotype and cognition in 221,124 middle- to older-aged (mean age 56.2 ± 8.0 years) women from the UK Biobank. Performance on six cognitive tasks was assessed, covering four cognitive domains: episodic visual memory, numeric working memory, processing speed, and executive function.

**Results:**

A longer reproductive span, older age at menopause, older age at first and last birth, and use of hormonal contraceptives were positively associated with cognitive performance later in life. Number of live births, hysterectomy without oophorectomy and use of hormone therapy showed mixed findings, with task-specific positive and negative associations. Effect sizes were generally small (Cohen’s *d* < 0.1). While *APOE* ε4 genotype was associated with reduced processing speed and executive functioning, in a dose-dependent manner, it did not influence the observed associations between female-specific factors and cognition.

**Discussion:**

Our findings support previous evidence of associations between a broad range of female-specific factors and cognition. The positive association between a history of hormonal contraceptive use and cognition later in life showed the largest effect sizes (max. *d* = 0.1). More research targeting the long-term effects of female-specific factors on cognition and age-related neurocognitive disorders including Alzheimer’s disease is crucial for a better understanding of women’s brain health and to support women’s health care.

## Introduction

Numerous age-related neurocognitive disorders show prominent sex and gender differences in their prevalence and presentation. For instance, relative to men, women are more likely to develop Alzheimer’s disease (AD), and are afflicted with poorer cognitive outcomes (Laws et al., 2018). Women’s health, however, has historically been understudied, and little is known about the roles of female-specific factors, such as reproductive history and hormone use, for cognitive performance later in life (Taylor et al., 2019).

Emerging epidemiological evidence suggests a positive association between reproductive span (age at menarche until age at menopause) and global and domain-specific cognition later in life (Ryan et al., 2009; Heys et al., 2011; Karim et al., 2016; Li et al., 2016; Song et al., 2020). However, other studies found no such associations (Low et al., 2005; Ryan et al., 2009). Age at menarche (Ryan et al., 2009; Karim et al., 2016) and age at menopause (McLay et al., 2003; Kuh et al., 2018) have each been both negatively and positively associated with cognitive functioning. While a later natural transition to menopause [on average at 51.4 years (Brinton et al., 2015)] may positively influence late life cognition, a recent systematic review suggests an association between surgical menopause, i.e., the removal of both ovaries (bilateral oophorectomy) before the onset of menopause, and faster cognitive decline in several cognitive domains (Puri et al., 2001). Similarly, a hysterectomy (removal of the uterus), often combined with bilateral oophorectomy, may also impact cognitive performance later in life (Puri et al., 2001). However, results are mixed.

Another factor which may alter both the length of the reproductive span (Mishra et al., 2017) and cognition is a history of childbirths. A higher number of childbirths may extend the length of a women’s reproductive span (Dorjgochoo et al., 2008), and has been linked to positive outcomes in cognitive domains such as processing speed, visual memory (Ning et al., 2020), and verbal memory (Orchard et al., 2020). However, other studies found no such associations (Ryan et al., 2009; Karim et al., 2016), or *negative* associations (McLay et al., 2003; Heys et al., 2011; Song et al., 2020). These discrepancies between findings may be linked to potential non-linear effects (Li et al., 2016; Ning et al., 2020). Compared to having two children, both no or one child and three or more children have been associated with poorer performance in verbal memory and executive function (Read and Grundy, 2017). Besides the number of childbirths, maternal age at first and last birth might further modulate late life cognition (Ryan et al., 2009; Read and Grundy, 2017).

In addition to the associations of reproductive span and parity with late-life cognition, hormone uses in the form of hormonal contraception (HC) to commonly prevent pregnancies or hormone therapy (HT) to alleviate menopausal symptoms may further modulate women’s late-life cognitive performance. Despite some studies to the contrary (Kang and Grodstein, 2012), the use of HT has largely been associated with protective effects on cognition across multiple domains, including verbal memory (Kampen and Sherwin, 1994; Zec and Trivedi, 2002), visual memory (Ryan et al., 2009), and global cognition (Song et al., 2020). However, this positive association between HT use and cognition may be modulated by genotype, age at initiation and duration of use. Carried by 14% of the world’s population, the apolipoprotein E type 4 (*APOE* ε4) allele is a known risk factor for AD (Eisenberg et al., 2010). Yaffe et al. (2000) found that current HT use reduced the risk of lower cognitive performance by almost half compared to never-users, but only in non-carriers (Yaffe et al., 2000). Similarly, results from the Nurses’ Health Study found that HT use was associated with faster decline in general cognition, especially among women with an *APOE* ε4 allele (Kang and Grodstein, 2012). Furthermore, according to the “*critical window hypothesis*,” the use of HT may be beneficial for cognition when initiated during perimenopause, while potentially detrimental if initiated later (Maki, 2013). However, other studies did not support this hypothesis (Kang and Grodstein, 2012). Whether duration of HT use modulates cognition beyond or independent of an individual’s age at initiation is unclear.

Relative to HT, the influence of HC on cognition is far less studied, despite its widespread use (Taylor et al., 2019). Only a limited number of studies have investigated the association between HC use and cognition later in life, with mixed results: some report positive correlations between HC use and global cognition and verbal memory in late life (Karim et al., 2016; Li et al., 2016; Song et al., 2020), while others do not (McLay et al., 2003; Ryan et al., 2009). Data from the Wisconsin Registry for Alzheimer’s Prevention suggests a positive association between HC duration and cognitive performance (Egan and Gleason, 2012). However, the sample of HC never-users was small (*n* = 34), precluding firm conclusions.

In summary, previous research indicates that reproductive history and hormone use may influence women’s cognition later in life. However, the findings have been inconclusive and both positive, negative and no associations have been reported, possibly due to a combination of small samples and various moderating factors. Here, we investigated the association of reproductive years, reproductive history, and HT and HC use with cognition in 221,124 middle- to older-aged women from the UK Biobank. Based on previous studies (Heys et al., 2011; Karim et al., 2016; Read and Grundy, 2017; Kuh et al., 2018; Ning et al., 2020), we assumed that female-specific factor may be particularly associated with memory, executive function and processing speed. To examine the effect of *APOE* ε4 genotype status, which has been suggested to modify the associations between female-specific factors and cognition, follow-up analyses including interaction terms were performed for each of the measures of interest.

## Methods and materials

### Participants

The sample was drawn from the UK Biobank cohort,^1^ and included 273,384 women. The UK Biobank acquired biological sex as binary variable from the central registry at recruitment and participants were able to update their recorded sex. While we acknowledge that individuals who are biologically female do not always identify as women, we used the gendered term “women,” where appropriate, to account for societal factors contributing to reproductive history, hormone use and cognition, such as access to childcare, healthcare and education. To ascertain a cognitively healthy sample, participants with diagnosed brain disorders known to influence cognition were excluded from the main sample (*n* = 39,011, see Supplementary Note S1), as were participants who later withdrew their consent (*n* = 6). Sample demographics of participants with sufficient data on key demographic variables including age, education, ethnicity, Townsend deprivation index and lifestyle score, amounting to 221,124, are provided in Table 1. Sample demographics stratified by HT and HC user status are displayed in Supplementary Tables S1, S2, respectively, and sample demographics stratified by presence or absence of a history of bilateral oophorectomy and/or hysterectomy are stated in Supplementary Table S3.

**Table 1:** Sample demographics.

**Total N**	**221,124**
Age (years)*	56.2 ± 8.0
Age range (years)	39–70
Education, N (%)	
College/University degree	73,577 (33.5)
O levels/GCSEs or equivalent	52,103 (23.7)
None of the above	33,165 (15.1)
A levels/AS levels or equivalent	27,167 (12.4)
Other professional qualifications	12,764 (5.8)
CSEs or equivalent	11,499 (5.2)
NVQ/HND/HNC or equivalent	9,307 (4.2)
Ethnic background, *N* (%)	
White	210,498 (95.2)
Asian	3,298 (1.5)
Black	3,282 (1.5)
Chinese	791 (0.4)
Other ethnic groups	1,849 (0.8)
Mixed	1,406 (0.6)
Townsend Deprivation Score*	−1.5 ± 2.9
Lifestyle score*	2.9 ± 1.6

*Continuous data in mean ± standard deviation and categorical data as number (%). N, number; O, ordinary level qualification; GCSE, general certificate of secondary education; A, advanced level qualification; AS, advanced subsidiary level qualification; CSE, certificate of secondary education; NVQ, national vocational qualification; HND, higher national diploma; HNC, higher national certificate.

### Cognitive assessment

Six computerized cognitive tests that were completed by more than 10% of the whole study population at baseline were selected. The included tests covered 4 cognitive domains, namely *visual memory* (Pairs Matching Test, *N* = 220,084, number of errors), *working memory* (Digit Span Test, *N* = 22,184, number of correct digits), *processing speed* (simple - Reaction Time Test, *N* = 220,098, in milliseconds; complex - Symbol Substitution Test, *N* = 58,083, number of correct substitutions), and *executive function* (Trail Making Test A and B, *N* = 50,464, in deciseconds). The cognitive tests are described in detail elsewhere (Lyall et al., 2016; Fawns-Ritchie and Deary, 2020). All test scores, except for the symbol digit test, were log-transformed. As the raw scores for the pair matching test and digit span test included zero values, plus one was added before transformation. For reaction time, potential outliers with scores below 50 milliseconds and above 2,000 milliseconds were removed. For the Symbol Digit Test, scores below three and above 36 were removed as outliers. To ease interpretation of results, Reaction Time, Trail Making A and B, as well as Pair Matching scores were inverted (multiplied by −1) so that higher scores on all the cognitive tests reflect better performance.

### Assessment of female-specific factors

Female-specific factors included length of reproductive span in years (age at menopause - age at menarche), age at menopause, age at menarche, number of live births, age at first and last childbirth, history of pregnancy loss during first trimester (miscarriage/termination) and after the second trimester (stillbirth), history of and age at bilateral oophorectomy and/or hysterectomy, HT and HC usage status (current-user/past-user/never-user), and duration of use and age at initiation among the users (for details see Supplementary Table S4). Participants who had missing data, or had responded “do not know,” “prefer not to answer,” “none of the above” or similar for each of the relevant variables, were excluded for the respective analyses.

### Genotyping

To assess *APOE* genotype, we used the extensively quality-controlled UK Biobank version 3 imputed data (Bycroft et al., 2018). *APOE* ε genotype was approximated based on two *APOE* ε single-nucleotide polymorphisms—rs7412 and rs429358, in accordance with previous work (Lyall et al., 2016). APOE ε4 status was labeled *carrier* for ε3/ε4 and ε4/ε4 combinations, and *non-carrier* for ε2/ε2, ε2/ ε3, and ε3/ ε3 combinations (Lyall et al., 2020). To test for potential dose-dependent effects, ε3/ε4 was labeled carrier of one ε4 allele, and ε4/ε4 as carrier of two ε4 alleles. Due to its ambiguity with ε1/ε3, the homozygous ε2/ε4 allele combination was removed (Seripa et al., 2007).^2^ Sample demographics stratified by *APOE* ε4 status are displayed in Supplementary Table S5.

### Statistical analysis

Multiple linear regression analyses were performed to investigate the relationship between female-specific factors (independent variable) and cognitive test scores (dependent variable). All models included the following additional independent variables known to influence reproductive history, hormone use and cognition: age, education, body mass index (BMI), Townsend deprivation index, and lifestyle score (Park and Reuter-Lorenz, 2009; Lovden et al., 2020; Boyle et al., 2021; see Supplementary Note S2 for details on Townsend deprivation index and lifestyle score). In addition, the analyses for reproductive span and age at menopause were corrected for use of HT, use of HC, history of bilateral oophorectomy and/or hysterectomy, and number of live births. We also investigated potential non-linear effects of the number of childbirths on cognition, by adding a quadratic term to the model, and by adding number of childbirths (0, 1, 2, 3, 4, 5, 6, and 7–22) as a dummy variable instead of a continuous variable. In latter model, participants with zero childbirths served as a reference group. Lastly, additional multiple linear regression models were fitted including an *APOE* ε4 status × female-specific factor interaction term to assess the associations between *APOE* ε4 status and female-specific factors on cognition. The models were adjusted for the same covariates as listed above. Furthermore, we tested for main effects of APOE ε4 status on cognitive performance, again adjusting for the same covariates.

**Figure 1:**
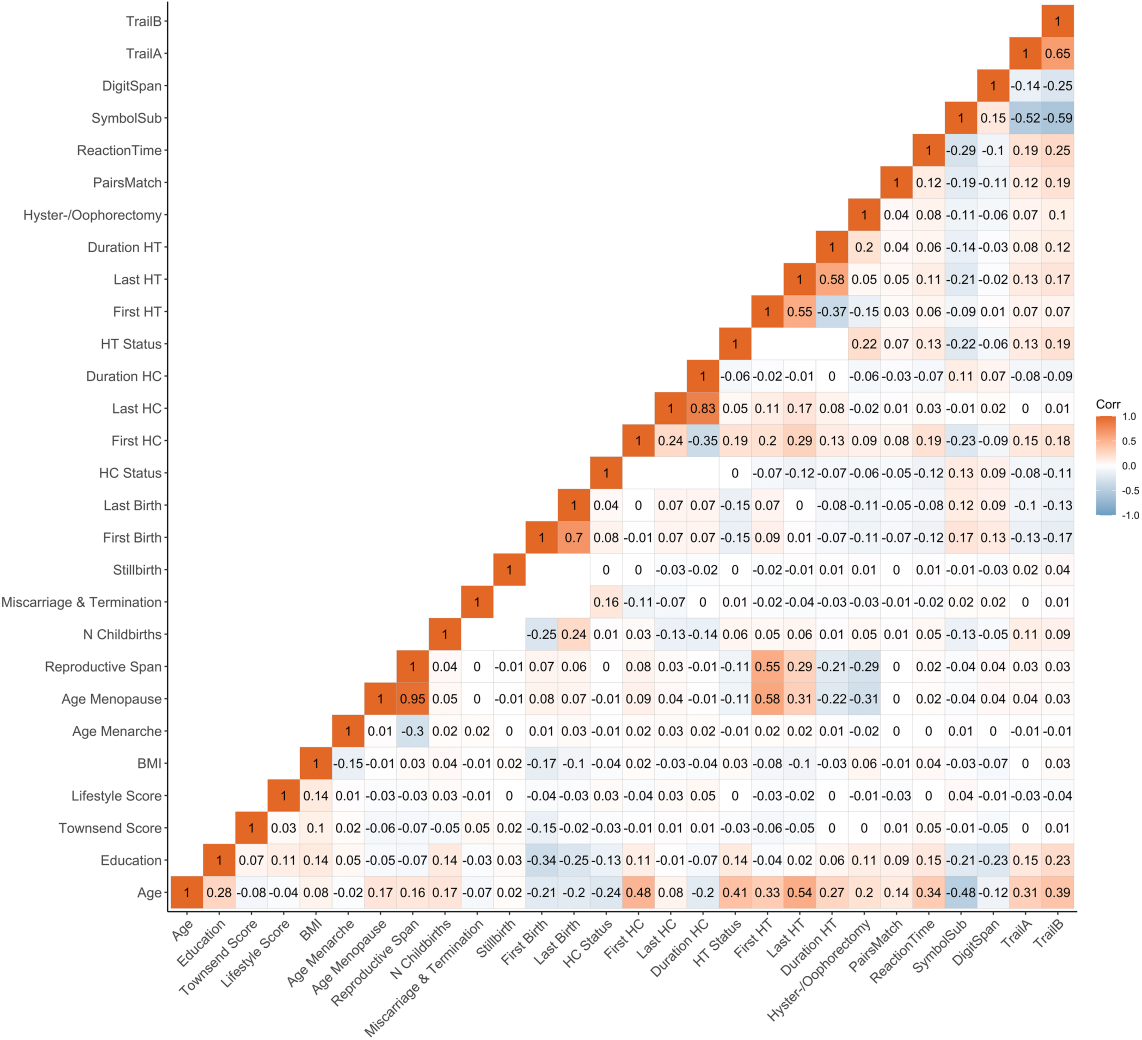
Correlations (Pearson’s r) between demographics, female-specific factors and cognitive test scores. Correlation between each pair of variables is computed using all complete pairs of observations on those variables. Empty fields indicate no complete pairs for that pair of variables. BMI, body mass index; HT, hormone therapy; HC, hormonal contraceptive; N, number; First Birth, age at first live birth (years); Last Birth, age at last live birth (years); First HC, age at first HC use (years); Last HC, age at last HC use (years); First HT, age at first HT use (years); Last HT, age at last HT use (years).

The statistical analyses were conducted using R, version 4.1.2 (R Core Team, 2022). All variables were standardized (subtracting the mean and dividing by the SD) prior to the regression analyses. To account for multiple comparisons, the *p*-values are reported after correcting for false discovery rate (p_FDR_, across all main models). The significance threshold was set to α = 0.05. We computed the Cohen’s *d* effect sizes from the *t*-statistics for categorical variables and *via* the partial correlation coefficient (*r*) for continuous variables (Nakagawa and Cuthill, 2007). Correlations between demographics, female-specific factors and cognitive tests are shown in Figure 1.

### Sensitivity analyses

To control for the potential influence of extreme values on our results, we assessed each continuous female-specific factor for outliers and excluded the corresponding participants before re-running the respective main analysis. To identify extreme values, we applied the median absolute deviation (MAD) method, implemented in the R package *Routliers*,^3^ using default settings (i.e., MAD threshold of ±3). This approach has the advantage of being robust with respect to sample size and the presence of extreme values. Furthermore, the main models were re-run (1) including previously excluded participants with diagnosed brain disorders to test whether results are sensitive to potential selection biases, and (2) also adjusting for *APOE* ε4 status.

According to the “healthy cell bias” hypothesis, estrogen exposure at an older age may lead to worse brain outcomes due to its neurotoxic effects in already damaged cells (Brinton, 2008). Hence the association between HT use and HC use and cognition may vary by participant’s health status. Hence, we rerun the respective analyses excluding participants with diagnosed illnesses suspected to be influenced by sex hormone exposure, i.e., autoimmune diseases (Lateef and Petri, 2012; *n* = 20,094), metabolic disorders (Salpeter et al., 2006; Korljan et al., 2010; *n* = 14,368) and cancer (*n* = 19,842), in addition to the already excluded participants with diagnosed brain disorders (see Supplementary Table S6 for corresponding ICD-10 diagnoses).

## Results

**Figure 2:**
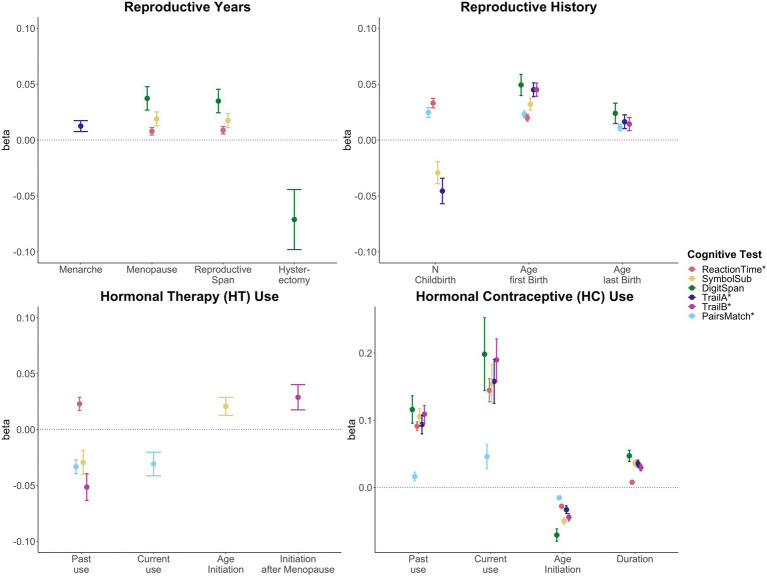
Significant associations between female-specific factors and cognitive performance. Point plot of beta-values with standard error from separate multiple regression analysis with cognitive task as dependent variable and female-specific variables as independent variable. All models are adjusted for age, education, body mass index, Townsend deprivation score, and lifestyle score. In addition, the analyses for reproductive span and age at menopause were corrected for use of HT, use of HC, history of hysterectomy and bilateral oophorectomy, and number of live births. The HT models were additionally adjusted for history of hysterectomy and bilateral oophorectomy, and the hysterectomy/oophorectomy model was co-varied for use of HT. All variables were standardized prior to performing the multiple linear regression analysis (subtracting the mean and dividing by the standard deviation). For visualization purposes, cognitive tests marked with * are inverted (multiplied by −1) so that positive beta-values always indicate associations between higher values on the female-specific variables and better performance on cognitive tests.

Significant associations between female-specific factors and cognitive performance are displayed in Figure 2. All tested associations are visualized in Supplementary Figure S1; Supplementary Table S7.

### Reproductive years and cognitive performance

A longer *reproductive span* was associated with faster processing speed (Reaction Time: β = 0.009, *p* = 0.009, Cohen’s *d* = 0.017; Symbol Substitution: β = 0.017, p_FDR_ = 0.016, *d* = 0.035), and higher working memory scores (Digit Span: β = 0.035, p_FDR_ = 0.003, *d* = 0.066). Similar positive associations were found for *age at menopause*. However, older *age at menarche* was associated with higher executive function scores (Trail Making A: β = 0.013, p_FDR_ = 0.028, *d* = 0.025).

While a history of hysterectomy, without bilateral oophorectomy, was associated with lower working memory scores (Digit Span: β = −0.071, p_FDR_ = 0.025, *d* = −0.071), bilateral oophorectomy, as a proxy of surgical menopause, was not significantly associated with cognitive performance, after FDR correction. Similarly, after correction, *age at bilateral oophorectomy* and/or *age at hysterectomy* were not significantly associated with cognitive scores.

### Reproductive history and cognitive performance

A higher *number of live childbirths* was associated with faster simple processing speed (Reaction Time: β = 0.033, p_FDR_ = 1.03e-13, *d* = 0.037), and higher visual memory scores (Pair Matching: β = 0.025, p_FDR_ = 1.05e-07, *d* = 0.027). However, a higher number of live childbirths was also associated with slower complex processing speed (Symbol Substitution: β = −0.029, p_FDR_ = 0.010, *d* = −0.027) and lower executive functioning scores (Trail Making A: β = −0.046, p_FDR_ = 2.68e-04, *d* = −0.039).

**Figure 3:**
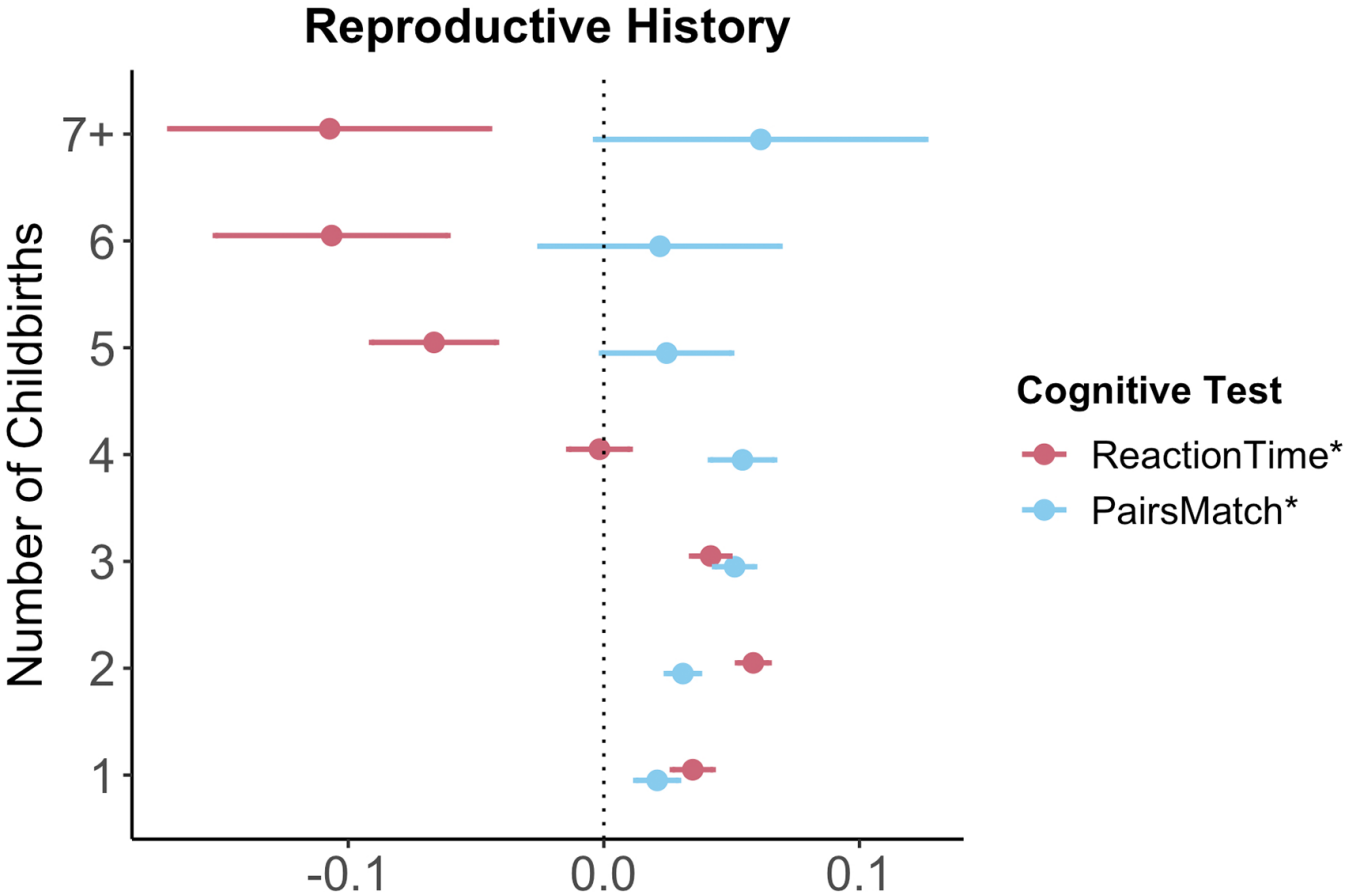
Significant non-linear association between number of childbirths and cognitive performance. Point plot of beta values with standard error from multiple regression analyses with cognitive task as dependent variable and number of childbirths as categorical independent variable. All models are adjusted for age, education, body mass index, Townsend deprivation score, and lifestyle score. All variables were standardized prior to performing the multiple linear regression analysis (subtracting the mean and dividing by the standard deviation). For visualization purposes, cognitive tests marked with * are inverted (multiplied by −1) so that positive beta-values always indicate associations between higher values on the female-specific variables and better performance on cognitive tests. Sample size for Pair Matching (0 = 34,881, 1 = 23,387, 2 = 77,687, 3 = 30,478, 4 = 7,596, 5 = 1,569, 6 = 446, 7+ = 234). Sample size for Reaction Time (0 = 34,699, 1 = 23,232, 2 = 77,308, 3 = 30,262, 4 = 7,520, 5 = 1,545, 6 = 430, 7+ = 226).

For visual memory and simple processing speed, we found a significant non-linear association with the number of live childbirths. Follow-up multiple linear regression models including number of live childbirths as a categorical variable showed that up to three and four childbirths were associated with faster simple processing speed and higher visual memory scores, respectively. More than four childbirths were associated with slower simple processing speed (Figure 3; Supplementary Table S8). In parous individuals, an older *age at first childbirth* was associated with higher cognitive performance scores on all six tests. Similarly, older *age at last childbirth* was associated with higher scores for visual memory, working memory and executive functioning.

Relative to nulliparous individuals, a *history of pregnancy loss* during first (miscarriage/termination) and after the second trimester (stillbirth), without a history of live births, was not associated with cognitive performance, after FDR-correction.

### Hormonal contraception and cognitive performance

*Past HT use* was associated with faster simple processing speed (Reaction Time: β = 0.023, p_FDR_ = 3.79e-04, *d* = 0.036), but slower complex processing speed (Symbol Substitution: β = −0.029, p_FDR_ = 0.018, *d* = −0.046). Past HT use was also associated with lower executive functioning scores (Trail Making B: β = −0.051, p_FDR_ = 7.56e-05, *d* = −0.048). Both *past* and *current HT use* were associated with lower visual memory scores (Pair Matching; *past use*: β = −0.033, p_FDR_ = 3.64e-07, *d* = −0.048; *current use*: β = −0.031, p_FDR_ = 0.012, *d* = −0.026). While *duration of HT use* was not significantly associated with any of cognitive tests, an older *age at HT initiation* was associated with faster complex processing speed (Symbol Substitution: β = 0.021, p_FDR_ = 0.027, *d* = 0.042). We further tested whether *age at HT initiation relative to age at menopause* (age started HT – age at menopause) was associated with late life cognition. *HT initiation after the onset of menopause* was associated with higher executive function scores (Trail Making B: β = 0.029, p_FDR_ = 0.028, *d* = 0.054).

### Hormone therapy and cognitive performance

*Current and past use of HC* were significantly associated with higher scores on all six cognitive tests. A longer *duration of HC use* was significantly associated with higher performance score in five of the six cognitive tests, except visual memory. An *older age at HC initiation* was associated with lower performance scores in all cognitive tests assessed.

### Sensitivity analyses

Most results were robust after either (1) removing extreme values (Supplementary Table S9), (2) including previously excluded participants with known brain disorders (Supplementary Table S10), and (3) additionally adjusting for *APOE* ε4 status (Supplementary Table S11), with slight variations. Similarly, HC and HT-related analyses were robust after removing additional diagnoses suspected to be influenced by sex hormone exposure (Supplementary Table S12). Detected extreme values are highlighted in Supplementary Table S13.

### *APOE* ε4 genotype and cognitive performance

**Figure 4:**
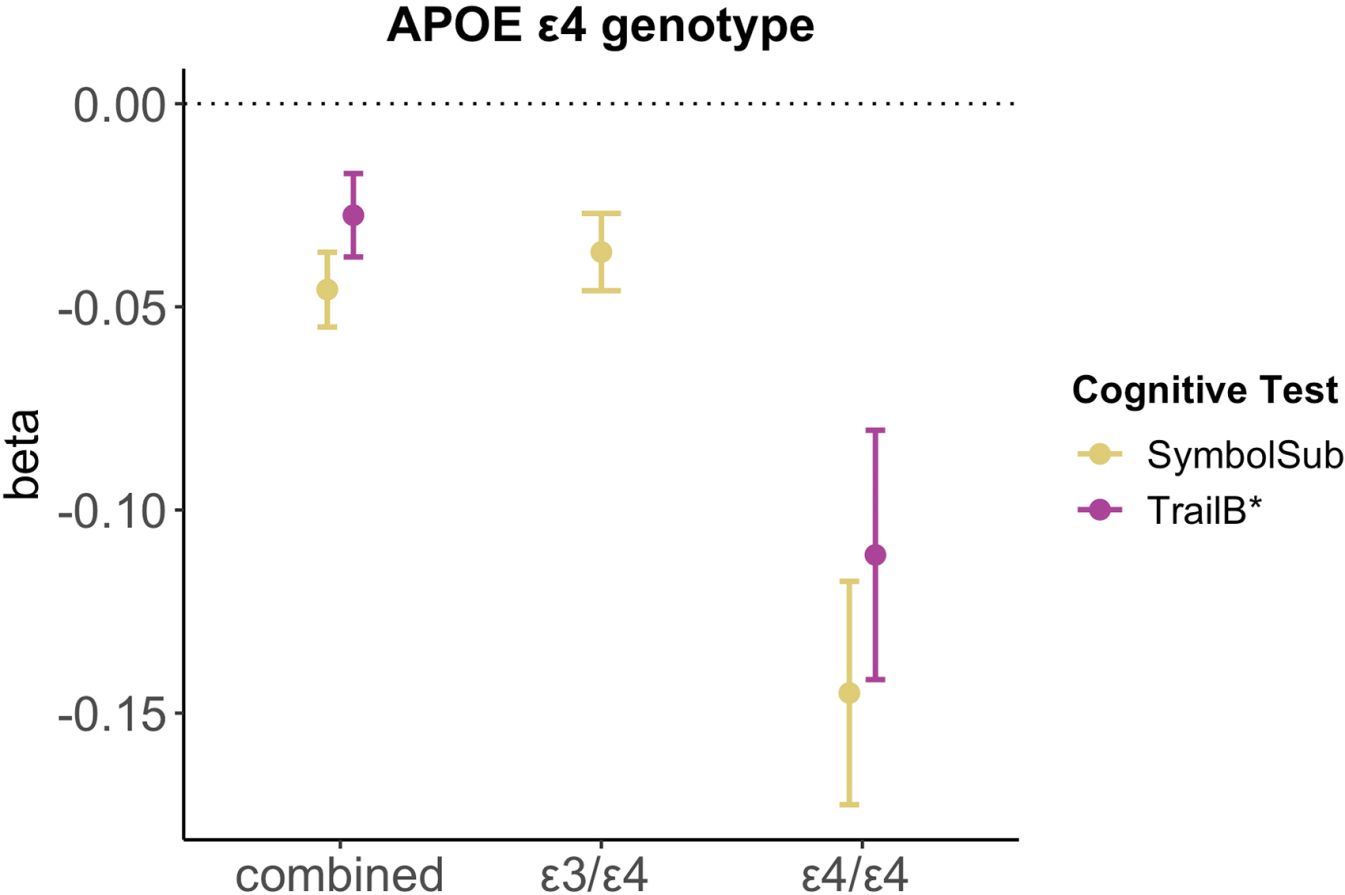
Significant associations between *APOE* ε4 genotype and cognitive performance. Point plot of beta-values with standard error from multiple regression analyses with cognitive task as dependent variable and APOE ε4 genotype as categorical independent variable. All models are adjusted for age, education, body mass index, Townsend deprivation score, and lifestyle score. All variables were standardized prior to performing the multiple linear regression analysis (subtracting the mean and dividing by the standard deviation). For visualization purposes, cognitive tests marked with * are inverted (multiplied by −1). Sample size for Symbol Substitution (non-carrier = 34,266, carrier one e4 allele = 11,023, carrier two e4 allele = 1,028) and Trail Making B (non-carrier = 29,865, carrier one e4 allele = 9,559, carrier two e4 allele = 893).

Relative to non-carriers, carriers of *APOE* ε4 alleles showed significantly lower executive functioning scores (Trail B: β = −0.028, p_FDR_ = 0.027, *d* = −0.030) and slower complex processing speed (Symbol substitution: β = −0.046, p_FDR_ = 5.93e-06, *d* = −0.053). Follow-up analyses suggest dose-dependent effects of *APOE* ε4 genotype on executive functioning (Trail B: two ε4 alleles β = −0.111, p_FDR_ = 0.001, *d* = −0.107; one ε4 allele β = −0.020, p_FDR_ = 0.123, *d* = −0.055), with carriers of two ε4 alleles, not one allele, showing lower cognitive performance scores (see Figure 4). Relative to non-carriers, slower complex processing speed was found in carriers with one and two ε4 alleles (Symbol substitution: two ε4 alleles β = −0.145, p_FDR_ = 2.39e-06, *d* = −0.057; one ε4 allele β = −0.037, p_FDR_ = 7.39e-04, *d* = −0.042, see Supplementary Table S14).

We found no significant interactions between *APOE* ε4 genotype and female-specific factors on cognition (Supplementary Table S15), except for number of live childbirths as well as number of live childbirths squared and simple processing speed (Reaction time: N Childbirths β = 0.039, p_FDR_ = 0.022, N Childbirths (Taylor et al., 2019) β = −0.046, p_FDR_ = 0.017). However, after excluding extreme value (number of childbirths >6), the interaction did not survive correction for multiple comparison.

## Discussion

The results of the present study indicate that a longer reproductive span, older age at menopause, older age at first and last childbirth, and use of hormonal contraceptives (HC) are positively associated with cognition later in life. Number of live births, hysterectomy without bilateral oophorectomy, and use of hormone therapy showed mixed findings, with task-specific positive and negative associations. Most of the results remained significant after additional sensitivity analyses. While effect sizes were generally small (*d* < 0.1), the association between a history of HC use and cognition later in life showed the largest effect sizes (max. *d* = 0.1).

A longer reproductive span with an older age at menopause was associated with better simple processing speed, complex processing speed, and numeric working memory. Older age at menarche was also associated with faster complex processing speed later in life. Our results align with a number of previous studies suggesting a positive association between reproductive span and cognition (Ryan et al., 2009; Heys et al., 2011; Karim et al., 2016; Li et al., 2016; Song et al., 2020). Studies on the effect of age at menarche on late-life cognition are inconclusive (Ryan et al., 2009; Karim et al., 2016; Song et al., 2020), and may be confounded by an inaccurate recall of age at menarche (Koo and Rohan, 1997). Several previous studies found a positive association between higher age at menopause and cognition (McLay et al., 2003; Kuh et al., 2018; Song et al., 2020).

A history of hysterectomy, without bilateral oophorectomy, was associated with lower working memory scores. Bilateral oophorectomy, as proxy of surgical menopause, and age at hysterectomy and/or bilateral oophorectomy were not associated with cognitive performance later in life. While some studies reported no significant associations between cognition and proxies of surgical menopause (McLay et al., 2003; Low et al., 2005; Ryan et al., 2009), a recent systematic review associated surgical menopause at any age with a faster decline in verbal and semantic memory, and processing speed (Georgakis et al., 2019). However, the definition of surgical menopause varied across studies, rendering firm conclusions difficult. In general, our findings for reproductive years align with another study using data from UK Biobank, showing that a shorter reproductive span, younger age at menopause and a history of hysterectomy were associated with greater risk for all-cause dementia (Gong et al., 2022). Similarly, a shorter reproductive span with early age at natural or surgical menopause has also been linked to a higher risk of incident cardiovascular disease (Ley et al., 2017).

The observed positive association between longer reproductive span and better late-life cognition might be understood through the protective role of estradiol, the most prevalent and potent estrogen in the female body (Thomas and Potter, 2013). Menopause is characterized by a cessation of ovarian function and a subsequent estradiol withdrawal. One might speculate that a longer reproductive span with an older age at menopause results in a higher life-time exposure to estradiol, which has been linked to positive effects on cognition (Al-Azzawi, 1992). Yet, female’s estradiol exposure is modulated, among others, by their reproductive history (Taylor et al., 2019; Cardenas et al., 2020), and the impact of parity on cognition is inconclusive. Of note, estradiol withdrawal during the transition to menopausal has been linked to rises in chronic low-grade inflammation as well as neurological and metabolic changes, which seem to promote a number of debilitating symptoms including hot flashes and night sweats, as well as mood, sleep, and cognitive disturbances (Brinton et al., 2015). The UK Biobank dataset does not include information on putative menopausal symptoms at the time of cognitive testing, which could have impacted participant’ cognitive performance (Conde et al., 2021).

We found that a higher number of live births was associated with both *better* visual memory scores and faster simple processing speed, and *slower* complex processing speed and lower executive functioning scores. In addition, we showed a non-linear relationship between number of live childbirths and simple processing speed as well as visual memory: having two to three live births was associated with better late life cognition than having one and more than four. The positive effects of parity on visual memory and simple processing speed corroborate previous results (Ning et al., 2020; Orchard et al., 2020). Similarly, a number of studies in rodents also showed positive associations between parity and memory performance (Nilsen and Brinton, 2002; Duarte-Guterman et al., 2019). While a number of studies suggest a non-linear effect of number of childbirths on cognition (Li et al., 2016; Read and Grundy, 2017) and all-cause dementia (Gong et al., 2022), other studies did not replicate these associations (Ning et al., 2020). Further studies are needed to confirm if any positive effect of parity on late-life cognition may be less pronounced in single- and grand-parous individuals.

Older age at first and last childbirth was associated with better cognitive functioning in multiple domains. While these findings are in line with several studies (Ryan et al., 2009; Read and Grundy, 2017), previous reports have been mixed (Karim et al., 2016; Song et al., 2020). In the current study, older age at first and last birth was correlated with higher education levels (see Figure 1). This is in line with previous research highlighting that older motherhood has been linked to higher socioeconomic status, total income, educational attainment, labor force participation and lower total number of childbirths (Barnes, 2001; Tearne, 2015). Hence, although we account for socioeconomic factors and education in our models, our results may be influenced by the great number of psychosocial variables associated with older motherhood and cognition.

A meta-analysis on the impact of pregnancy on memory function found that both pregnant and postpartum individuals showed lower scores in memory measures that place high demands on executive cognitive control (Henry and Rendell, 2007). Yet, it is unclear how long these domain-specific cognitive disturbances persist. Here, we found that complex processing speed and executive functioning scores were lower in middle-to-older aged participants with a history of live births, suggesting that these cognitive functions may be altered long after the last birth.

The differential relationship between parity and cognition might be understood through a number of potential physiological and psychosocial mechanisms (Cardenas et al., 2020). First, pregnancy-related variations in estradiol might exert effects on cognition. Estradiol increases 300-fold during pregnancy, drops rapidly postnatally, and remains lower in parous individuals compared to nulliparous individuals all through menopause (Cardenas et al., 2020). Higher levels of estradiol have been linked to better visual memory (Rentz et al., 2017). Hence, lower estradiol levels in parous individuals might explain the observed negative association between parity and cognition. Second, the *“pregnancy compensation hypothesis”* suggests that a history of childbirths has a dampening effect on the otherwise ramped up female immune system throughout adulthood (Al-Azzawi, 1992). In this view, if pregnancies fail to occur, the immune system becomes overly activated and starts releasing auto-antibodies that attack healthy cells (Natri et al., 2019), giving rise to autoimmune diseases and AD. In line with immunologic effects, more time spent pregnant in the first trimester has been associated with lower risk for AD (Fox et al., 2018). This may explain the apparent positive effects of parity on cognition. However, we did not find an association between pregnancy loss during the first and after the second trimester and late life cognitive performance. Third, pregnancy and postpartum have been associated with heightened neuroplasticity to facilitate maternal behavior and caring for the offspring (Barba-Muller et al., 2019; Cardenas et al., 2020). For instance, better simple processing speed and visual memory in parous individuals may be behavioral adaptations to quickly respond to the needs of the child and to detect potential threads through enhanced associative memory encoding and retrieval, respectively. However, one might also speculate that being on alert to protect the child will reduce the capacity for mental flexibility, a part of executive function (Diamond, 2013). Growing evidence suggests heightened plasticity in the maternal brain, increasing responsiveness to both negative and positive experiences (Kim, 2016). Hence, factors modulating maternal experiences such as environment, genetics, and personality traits, may be drivers for how the maternal brain changes.

Both current and past use of HT were associated with lower visual memory scores. Past use of HT was also associated with higher simple processing speed, but lower complex processing speed and executive functioning scores. Duration of HT use did not influence late life cognition. However, older age at HT initiation was associated with faster complex processing speed. While a large number of previous studies reported on positive effects of HT use on cognition (Yaffe et al., 2000; Zec and Trivedi, 2002; Ryan et al., 2009; Song et al., 2020), a review covering the same six cognitive tasks of the present study also found both positive and negative associations with HT use (Zec and Trivedi, 2002). The heterogeneity in results is likely driven by different study designs (e.g., observational vs. experimental), sample sizes and adjusted covariates (Zec and Trivedi, 2002). In addition, detailed information on HT formulation [i.e., estrogens only vs. estrogens plus progestins; type of estrogens (e.g., estradiol or estrone) and progestins (e.g., synthesized or micronized)], modes of administration (i.e., oral, transdermal or vaginal) and dosage is rarely available; all factors which might modulate HT effects on cognition (Maki and Sundermann, 2009; Berent-Spillson et al., 2015).

Furthermore, timing of HT initiation, *APOE* ε4 genotype and socioeconomic as well as lifestyle factors may also modulate the HT effect on late life cognition. We found higher complex processing speed with older age at HT initiation and higher executive functioning scores with HT initiation after menopause, which is not in line with the “*critical window hypothesis*,” stating that HT is most beneficial for cognitive performance when administered perimenopausal (Maki, 2013). Age at HT initiation is intrinsically linked to age at menopause (r = 0.58, Figure 1), as HT is prescribed to alleviate menopausal symptoms or replenish endogenous sex hormone levels after bilateral oophorectomy and/or hysterectomy. Our results suggest that both an older age at menopause and an older age at first HT use may be positively associated with complex processing speed later in life.

While we found a slower complex processing speed and lower executive functioning scores in *APOE* ε4 carriers relative to non-carriers, in line with previous work (Wisdom et al., 2011), we found no significant interaction effect between *APOE* ε4 genotype and HT use on cognition. The latter result is in line with some studies (Kang et al., 2004). Yet others found less cognitive decline amongst HT using non-carriers (Yaffe et al., 2000; Kang and Grodstein, 2012). As the cognitive tests varied between studies, one might speculate that the previously observed HT-genotype interaction may be domain-specific. Levels of socioeconomic deprivation, measured with the Townsend deprivation index, and lifestyle score differed between HT users and non-users (see Supplementary Table S1). According to the *healthy user bias*, individuals using HT tend to be healthier and better educated, particularly in observational studies (Matthews et al., 1996; Barrett-Connor and Laughlin, 2009). This was not the case in the current study, potentially explaining test-specific negative associations with HT use, even after confound correction.

Contrary to the HT findings, we found that current and past use of HC were associated with higher performance on all cognitive tasks assessed. Younger age at HC initiation and a longer duration of use were significantly associated with higher cognitive performance scores later in life. While most studies in premenopausal individuals commonly report on no or inconclusive associations between HC use and a variety of cognitive tasks (Warren et al., 2014), two studies in menopausal individuals found duration-dependent increases in cognitive performance in HC users compared to never-users, especially in users with over 10 or 15 years of use (Egan and Gleason, 2012; Karim et al., 2016). Our results corroborate these findings in middle-to older-aged individuals. Of note, some participants in the current study still used HC in older age, as old as 70 years (Supplementary Table S2). These participants might have used HC to alleviate menopausal symptoms, rather than for contraceptive reasons, which may have impacted our results (Hammond et al., 2001). Emerging evidence suggests that different synthetic HC analogues affect cognition differently (Warren et al., 2014). For instance, HC effects on visuospatial-performance might be dependent on the androgenicity of the added progestin. Antiandrogenic preparations were associated with lower performance scores, while older generation progestins in HC with higher androgenicity were linked to higher scores (Wharton et al., 2008). This finding highlights the importance of accounting for HC formulations, which is lacking in the present study. The mechanisms behind the positive associations with long-term HC and cognition are far from understood and warrant further exploration, especially as many women start using HCs at a young age and continue using it for decades.

To the best of our knowledge, the current work is one of the largest, comprehensive studies of the associations between female-specific factors, *APOE* ε4 genotype, and cognition. Large-scale population-based studies enable the identification of subtle effects that could go undetected in smaller samples, and are key to identify factors that may contribute to cognitive aging and risk for neurodegenerative disease. However, the cross-sectional and non-experimental nature of the presented data does not enable causal inference, and randomized controlled longitudinal studies are required to fully understand how female-specific factors influence women’s late life cognition. While the UK Biobank is an unprecedented large-scale open-access resource to study population health, it is a homogeneous cohort with regard to ethnic background (95.2% white) and is as such not representative of the general population.

Furthermore, the cognitive test battery was limited, tapping only selected cognitive domains and functions, and not all cognitive domains which have been previously associated with sex hormones were assessed (Gervais et al., 2017). More research with a broader and more fine-grained array of cognitive tests is needed to elucidate the effects of female-specific factors on women’s cognitive functioning.

As highlighted above, the current study lacked details on HT and HC formulation, mode of delivery (e.g., oral or transdermal), and dosage. Of note, HT and HC formulations and dosages have changed significantly over time. For instance, while present day HCs typically contain 15 to 35 μg of ethinyl estradiol and lower amounts of progestin, estrogen content was up to ~150 μg and 1 to 10 mg of progestin when HCs were first introduced in the 1960s (Hampson, 2020). Given the age range of the UK Biobank cohort at baseline, 40–70 years, considerable differences in HC and HT dosages by age when first used may be present, and results from the current study may not translate to other cohorts.

Furthermore, while we account for socioeconomic and lifestyle factors known to influence reproductive history, hormone use and cognition, it is possible that other factors such as childhood cognition, nutrition and physical/somatic health also influence the association between female-specific factors and late-life cognition (Kuh et al., 2005). In addition, the present work relies on self-reported data for hormone use and reproductive events, which might not always be reliable. Finally, while the UK Biobank has a wealth of female-specific variables, how these variables are recorded might not align with best practice standards. For instance, menopause is defined as the absence of a menstrual period for 1 year (12 months), but recorded in the UK Biobank based on whether the menstrual period has generally stopped (Supplementary Table S4). Moving forward, harmonized guidelines on how female-specific variables are recorded are essential.

In summary, this study provides evidence for associations between female-specific factors and late life cognition. Specifically, longer reproductive span, higher age at menopause, higher age at first and last birth, and use of HC were associated with better cognitive performance later in life. The results for number of live births, hysterectomy and/or bilateral oophorectomy and use HT were mixed, with both positive and negative associations. In future research, well-designed longitudinal studies assessing the relationship between sex hormone levels and cognition across hormonal transition periods such as pregnancy and perimenopause are warranted to develop a better understanding of women’s brain health.

## Data availability statement

Publicly available datasets were analyzed in this study. This data can be found at: https://www.ukbiobank.ac.uk.

## Ethics statement

The current study includes human participants and has been conducted using the UK Biobank resource under Application 27412. The UK Biobank has received ethics approval from the National Health Service National Research Ethics Service (ref 11/NW/0382). The participants provided their written informed consent to participate in this study.

## Author contributions

LRSL, CKT, and CB designed the study. LRSL, CB, and DvdM performed the data analysis. LRSL, CKT, A-MGdL, and CB interpreted the data. LRSL and CB drafted and finalized the manuscript. A-MGdL, IA, LTW, CKT, and DvdM critically revised the first draft and approved the final manuscript. All authors contributed to the article and approved the submitted version.

## Funding

While working on this study, the authors received funding from the Research Council of Norway (CT: #223273, #288083, #323951; LTW: #273345, #249795, #298646, #300768, #223273; and IA: #213700, #223273, #250358), the South-Eastern Norway Regional Health Authority (CKT: #2019069, #2021070, #500189; LTW: #2018076, #2019101; and IA: #2017097, #2019104, #2020020), the European Research Council under the European Union’s Horizon 2020 Research and Innovation Program (LTW: #802998), and the Swiss National Science Foundation (A-MGdL: PZ00P3_193658).

## Conflict of interest

For work unrelated to the contents of this manuscript, IA received speaker’s honorarium from Lundbeck.

The remaining authors declare that the research was conducted in the absence of any commercial or financial relationships that could be construed as a potential conflict of interest.

## Publisher’s note

All claims expressed in this article are solely those of the authors and do not necessarily represent those of their affiliated organizations, or those of the publisher, the editors and the reviewers. Any product that may be evaluated in this article, or claim that may be made by its manufacturer, is not guaranteed or endorsed by the publisher.

## Footnotes

^1^ https://www.ukbiobank.ac.uk

^2^ https://www.snpedia.com/index.php/APOE

^3^ https://CRAN.R-project.org/package=Routliers
