# Supplementary material for: Associations between reproductive history, hormone use, APOE ε4 genotype and cognition in middle- to older-aged women from the UK Biobank
Source: Front Aging Neurosci. 2023 Jan 19;14:1014605. doi: 10.3389/fnagi.2022.1014605 (PMC9907169; doi:10.3389/fnagi.2022.1014605)
Supplement: Supplementary file 1 [file Data_Sheet_1.pdf]

SUPPLEMENTARY MATERIAL

Associations between reproductive history, hormone use, *APOE* ε4 genotype and cognition in middle- to older-aged women from the UK Biobank.

Linn R. S. Lindseth [1], Ann-Marie G. de Lange [2,3,4], Dennis van der Meer [1,5], Ingrid Agartz [1,6,7], Lars T. Westlye [3,8], Christian K. Tamnes [6,8,9] and Claudia Barth [1,6]*

[1] NORMENT, Institute of Clinical Medicine, University of Oslo, Oslo, Norway
[2] LREN, Department of Clinical Neurosciences, Centre for Research in Neurosciences, Lausanne University Hospital (CHUV) and University of Lausanne, Lausanne, Switzerland
[3] Department of Psychology, University of Oslo, Oslo, Norway
[4] Department of Psychiatry, University of Oxford, Oxford, United Kingdom
[5] School of Mental Health and Neuroscience, Faculty of Health, Medicine and Life Sciences, Maastricht University, Maastricht, Netherlands
[6] Department of Psychiatric Research, Diakonhjemmet Hospital, Oslo, Norway
[7] Department of Clinical Neuroscience, Centre for Psychiatry Research, Stockholm Health Care Services, Karolinska Institute, Stockholm County Council, Stockholm, Sweden
[8] NORMENT, Division of Mental Health and Addiction, Oslo University Hospital, Institute of Clinical Medicine, University of Oslo, Oslo, Norway
[9] Department of Psychology, PROMENTA Research Center, University of Oslo, Oslo, Norway

***Corresponding author:** Claudia Barth, PhD, E-Mail: claudia.barth@medisin.uio.no

Note 1: Participants with diagnosis known to influence cognition

Participants with diagnosed brain disorders known to influence cognition were excluded from the main sample (n = 39,011). Brain disorders were based on International Classification of Diseases (ICD)-10 diagnoses (chapter V and VI, field F; mental and behavioral disorders), including F00-F03 for Alzheimer's disease and dementia, and F06.7 (´Mild cognitive disorder`), field G (`Diseases of the nervous system´), including inflammatory and neurodegenerative diseases (except G55-59; ´Nerve, nerve root and plexus disorders´) and field I ('Diseases of the circulatory system'), including I64 for stroke. An overview of the diagnoses is provided in the UK Biobank online resources (https://biobank.ndph.ox.ac.uk/showcase/field.cgi?id=41270), and the diagnostic criteria are listed in the ICD10 diagnostic manual (https://www.who.int/classifications/icd/icdonlineversions).

Note 2: Townsend deprivation index and lifestyle score

The Townsend deprivation index was derived from national census data about car ownership, household overcrowding, owner occupation, and unemployment aggregated for postcodes of residence in the UK[1]. Higher scores reflect higher levels of socioeconomic deprivation. The lifestyle score was calculated based on sleep duration, time spent watching television, current and past smoking status, alcohol consumption frequency, physical activity level, intake of fruits and vegetables, and intake of oily fish, beef, lamb/mutton, pork and processed meat[1]. Each unhealthy lifestyle factor was scored with one point (e.g., smoking), and each participant's points were summed to generate an unweighted score (from 0-9): the higher the lifestyle score, the unhealthier the participant's lifestyle.

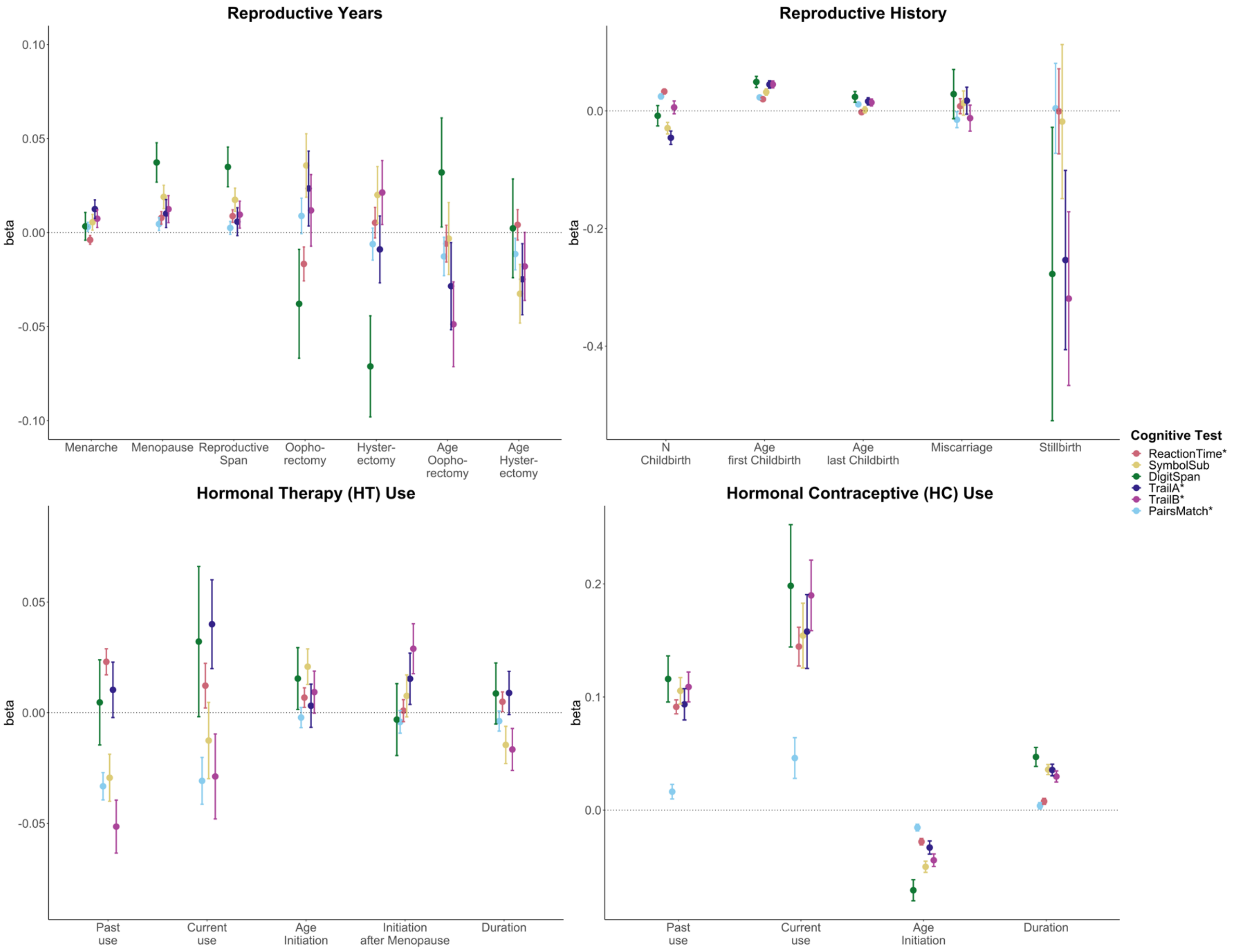
Reproductive Years
beta
0.10
0.05
0.00
-0.05
-0.10
Menarche
Menopause
Reproductive Span
Oopho- rectomy
Hyster- ectomy
Age Oopho- rectomy
Age Hyster- ectomy
Reproductive History
beta
0.0
-0.2
-0.4
N Childbirth
Age first Childbirth
Age last Childbirth
Miscarriage
Stillbirth
Hormonal Therapy (HT) Use
beta
0.05
0.00
-0.05
Past use
Current use
Age Initiation
Initiation after Menopause
Duration
Hormonal Contraceptive (HC) Use
beta
0.2
0.1
0.0
Past use
Current use
Age Initiation
Duration
Cognitive Test
ReactionTime*
SymbolSub
DigitSpan
TrailA*
TrailB*
PairsMatch*

Figure S1| Associations between female-specific factors and cognitive performance.

Point plot of beta-values with standard error from separate multiple regression analysis with cognitive task as dependent variable and female-specific variables as independent variable. All models are adjusted for age, education, body mass index, Townsend deprivation score, lifestyle score. In addition, the analyses for reproductive span and age at menopause were corrected for use of HT, use of HC, history of hysterectomy and bilateral oophorectomy, and number of live births. The HT models were additionally adjusted for history of hysterectomy and bilateral oophorectomy, and the hysterectomy/oophorectomy model was co-varied for use of HT. All variables were standardized prior to performing the multiple linear regression analysis (subtracting the mean and dividing by the standard deviation). For visualization purposes, cognitive tests marked with * are inverted (multiplied by -1) so that positive beta-values always indicate associations between higher values on the female-specific variables and better performance on cognitive tests.

Table S1| Sample demographics stratified by hormone therapy (HT) user groups.

	Never-User	Current-User	Past-User	p-value	test
**Total N**	138,963	12,663	60,083		
**Age** (years)*	53.5 ± 8.0	56.7 ± 6.3	61.2 ± 5.1	<0.001	KW
**Age range** (years)	39 – 70	40 – 70	40 – 70		
**Education**, N (%)				<0.001	χ2
College/University degree	51,475 (37.3)	4,343 (34.7)	16,020 (26.9)		
O levels/GCSEs or equivalent	32,214 (23.3)	3,017 (24.1)	14,620 (24.5)		
None of the above	15,542 (11.3)	1,580 (12.6)	12,965 (21.7)		
A levels/AS levels or equivalent	18,609 (13.5)	1,561 (12.5)	6,233 (10.5)		
Other professional qualifications	6,766 (4.9)	814 (6.5)	4,562 (7.7)		
CSEs or equivalent	8,120 (5.9)	673 (5.4)	2,320 (3.9)		
NVQ/HND/HNC or equivalent	5,337 (3.9)	537 (4.3)	2,912 (4.9)		
**Townsend Deprivation Score***	-1.5 ± 3.0	-1.6 ± 2.9	-1.7 ± 2.9	<0.001	KW
**Lifestyle score***	2.9 ± 1.6	3.0 ± 1.6	2.9 ± 1.6	<0.001	KW
**BMI** (kg/m^2)	26.7 ± 5.1	26.3 ± 4.5	27.1 ± 4.7	<0.001	KW
**Bilateral Oophorectomy**, Yes N (%)	3,151 (2.3)	3,135 (24.8)	9,458 (15.7)	<0.001	χ2
**Hysterectomy**, Yes N (%)	4,241 (3.2)	1,325 (14.9)	7,540 (15.2)	<0.001	χ2
**Age first used HT***[x] (years)		47.1 ± 5.9	47.8 ± 5.1	<0.001	KW
**Age last used HT***[x] (years)		56.6 ± 6.3	54.1 ± 5.7	<0.001	KW
**Duration HT use***[x] (years)		9.4 ± 6.8	6.3 ± 5.2	<0.001	KW

* Continuous data in mean ± standard deviation and categorical data as number (%).
[x] Complete data for 12,270 current-users and 58,467 past-users.
Abbreviations: N = Number; O = Ordinary Level Qualification; GCSE = General Certificate of Secondary Education; A = Advanced Level Qualification; AS = Advanced Subsidiary Level Qualification; CSE = Certificate of Secondary Education; NVQ = National Vocational Qualification; HND = Higher National Diploma; HNC = Higher National Certificate; KW = Kruskal-Wallis.

Table S2| Sample demographics stratified by hormonal contraceptive (HC) user groups.

	Never-User	Current-User	Past-User	p-value	test
**Total N**	39,908	4,322	160,392		
**Age** (years)*	60.0 ± 7.8	45.6 ± 4.0	55.3 ± 7.7	<0.001	KW
**Age range** (years)	39 – 70	40 – 70	40 – 70		
**Education**, N (%)				<0.001	$\chi 2$
College/University degree	11,215 (28.3)	1,742 (40.7)	56,262 (35.3)		
O levels/GCSEs or equivalent	8,695 (22.0)	1,100 (25.7)	38,212 (24.0)		
None of the above	9,794 (24.7)	116 (2.7)	19,761 (12.4)		
A levels/AS levels or equivalent	3,863 (9.8)	747 (17.4)	20,675 (13.0)		
Other professional qualifications	2,851 (7.2)	109 (2.5)	8,882 (5.6)		
CSEs or equivalent	1,440 (3.6)	333 (7.8)	8,844 (5.6)		
NVQ/HND/HNC or equivalent	1,737 (4.4)	136 (3.2)	6,680 (4.2)		
**Townsend Deprivation Score***	-1.3 ± 3.0	-1.3 ± 3.0	-1.6 ± 2.9	<0.001	KW
**Lifestyle score***	2.8 ± 1.6	3.1 ± 1.6	2.9 ± 1.6	<0.001	KW
**BMI** (kg/m^2)	27.2 ± 5.2	25.8 ± 4.7	26.8 ± 4.9	<0.001	KW
**Age first used HC***[x]		20.3 ± 5.6	21.5 ± 4.6	<0.001	KW
**Age last used HC***[x] (years)		45.6 ± 4.0	31.9 ± 7.5	<0.001	KW
**Duration HC use***[x]		25.2 ± 6.6	10.4 ± 7.7	<0.001	KW

* Continuous data in mean ± standard deviation and categorical data as number (%).
[x] Complete data for 4,219 current-users and 158,920 past-users.
Abbreviations: N = Number; O = Ordinary Level Qualification; GCSE = General Certificate of Secondary Education; A = Advanced Level Qualification; AS = Advanced Subsidiary Level Qualification; CSE = Certificate of Secondary Education; NVQ = National Vocational Qualification; HND = Higher National Diploma; HNC = Higher National Certificate; KW = Kruskal-Wallis.

Table S3| Sample demographics stratified by history of oophorectomy and/or hysterectomy.

**Total N**	**Women without Surgery** 166,278	**Women with Hysterectomy** 6,691	**Women with Bilateral Oophorectomy** 6,445	p-value	test
**Age** (years)*	55.4 ± 8.1	61.8 ± 5.5	61.1 ± 5.8	<0.001	KW
**Range** (years)	39-70	40-70	40-70		
**Education**, N (%)				<0.001	χ2
College/University degree	59,082 (35.8)	1,382 (20.8)	1,652 (25.9)		
O levels/GCSEs or equivalent	38,481 (23.3)	1,636 (24.6)	1,431 (22.4)		
None of the above	22,176 (13.4)	1,800 (27.1)	1,568 (24.5)		
A levels/AS levels or equivalent	20,980 (12.7)	623 (9.4)	615 (9.6)		
Other professional qualifications	9,165 (5.5)	498 (7.5)	497 (7.8)		
CSEs or equivalent	8,691 (5.3)	289 (4.3)	268 (4.2)		
NVQ/HND/HNC or equivalent	6,574 (4.0)	416 (6.3)	356 (5.6)		
**Townsend Deprivation Score***	-1.5 ± 2.9	-1.6 ± 2.9	-1.5 ± 3.0	0.003	KW
**Lifestyle score***	2.9 ± 1.6	2.8 ± 1.5	2.9 ± 1.6	0.001	KW
**BMI** (kg/m^2)	26.6 ± 4.9	27.5 ± 4.7	27.7 ± 5.2	<0.001	KW
**Menopause**, yes N(%)	112,191 (67.5)	6,523 (97.5)	6,381 (99.0)	<0.001	χ2
**Age at menopause*** (years)	50.4 ± 4.4	43.5 ± 6.9	47.4 ± 6.3	<0.001	KW
**Range** (years)	18-68	18-65	18-66		
**Age at hysterectomy*** (years)		44.8 ± 8.9	49.8 ± 8.0		
**Range** (years)		10-69	17-69		
**Age at bilateral oophorectomy*** (years)			50.2 ± 8.1		
**Range** (years)			11-69		
**Age at surgery relative to age at menopause, N (%)**					
Same Age		4,312 (66.2)	3,151 (49.5)		
After Age at Menopause		1,634 (25.1)	2,847 (44.7)		
Before Age at Menopause		567 (8.7)	366 (5.8)		

* Continuous data in mean ± standard deviation and categorical data as number (%).
Abbreviations: N = Number; O = Ordinary Level Qualification; GCSE = General Certificate of Secondary Education; A = Advanced Level Qualification; AS = Advanced Subsidiary Level Qualification; CSE = Certificate of Secondary Education; NVQ = National Vocational Qualification; HND = Higher National Diploma; HNC = Higher National Certificate; KW = Kruskal-Wallis.

Table S4| Definitions of female-specific variables in the UK Biobank

**Female specific variable**	**UKB Data-field**	**ACE touchscreen question**	**Coding**
Age at Menarche	2714	"How old were you when your periods started?"	Age in years<br>-1 («Do not know»)<br>-3 («Prefer not to answer»)
Menopause	2724	"Have you had your menopause (periods stopped)?"	1 ("Yes")<br>0 ("No")<br>2 («Not sure – had a hysterectomy»)<br>3 ("Not sure – other reason")<br>-3 («Prefer not to answer»)
Age at Menopause	3581	"How old were you when your periods stopped?"	Age in years<br>-1 («Do not know»)<br>-3 («Prefer not to answer»)
Oophorectomy	2834	"Have you had BOTH ovaries removed?"	1 ("Yes")<br>0 ("No")<br>-5 («Not sure»)<br>-3 («Prefer not to answer»)
Age at Oophorectomy	3882	"How old were you when you had BOTH ovaries removed?"	Age in years<br>-1 («Do not know»)<br>-3 («Prefer not to answer»)
Hysterectomy	3591	"Have you had a hysterectomy (womb removed)?"	1 ("Yes")<br>0 ("No")<br>-5 («Not sure»)<br>-3 («Prefer not to answer»)
Age at Hysterectomy	2824	"How old were you when you had your hysterectomy?"	Age in years<br>-1 («Do not know»)<br>-3 («Prefer not to answer»)
N Childbirth	2734	"How many children have you given birth to? (please include live births only)"	Number of children<br>-3 («Prefer not to answer»)
Age last Birth	2764	"How old were you when you had your LAST child?"	Age in years<br>-4 («Do not remember»)<br>-3 («Prefer not to answer»)
Age first Birth	2754	"How old were you when you had your FIRST child?"	Age in years<br>-4 («Do not remember»)<br>-3 («Prefer not to answer»)
Had Stillbirth, Miscarriage or Termination	2774	"Have you ever had any stillbirths, spontaneous miscarriages or terminations?"	1 ("Yes")<br>0 ("No")<br>-1 («Do not know»)<br>-3 («Prefer not to answer»)
N Stillbirths	3829	"How many stillbirths? (enter 0 if none)"	Number of stillbirths<br>-1 («Do not know»)

			-3 («Prefer not to answer»)
N Miscarriages	3839	"How many spontaneous miscarriages? (enter 0 if none)"	Number of miscarriages<br>-1 («Do not know»)<br>-3 («Prefer not to answer»)
N Terminations	3849	"How many terminations? (enter 0 if none)"	Number of miscarriages<br>-1 («Do not know»)<br>-3 («Prefer not to answer»)
HT use	2814	"Have you ever used hormone replacement therapy (HRT)?"	1 ("Yes")<br>0 ("No")<br>-1 («Do not know»)<br>-3 («Prefer not to answer»)
Age last HT use	3546	"How old were you when you last used HRT?"	Age in years<br>-1 («Do not know»)<br>-11 ("Still taking HRT")<br>-3 («Prefer not to answer»)
Age HT initiation	3536	"How old were you when you first used HRT?"	Age in years<br>-1 («Do not know»)<br>-3 («Prefer not to answer»)
HC use	2784	"Have you ever taken the contraceptive pill? (include the 'mini-pill')	1 ("Yes")<br>0 ("No")<br>-1 («Do not know»)<br>-3 («Prefer not to answer»)
Age last HC use	2804	"How old were you when you last used the contraceptive pill?"	Age in years<br>-1 («Do not know»)<br>-3 («Prefer not to answer»)<br>-11 ("Still taking the pill")
Age HC initiation	2794	"About how old were you when you first went on the contraceptive pill?"	Age in years<br>-1 («Do not know»)<br>-3 («Prefer not to answer»)

Abbreviation: N = Number, HT = Hormone Therapy, HC = Hormonal Contraceptive.

Table S5| Sample demographics stratified by APOE ε4 status.

	Non-Carrier	Carrier	p-value	test
**Total N**	**153,395**	**55,423**		
**Age** (years)*	57.0 [50.0, 63.0]	57.0 [50.0, 63.0]	<0.001	KW
**Education**, N (%)			0.039	χ2
College/University degree	51,463 (33.6)	18,497 (33.4)		
O levels/GCSEs or equivalent	36,225 (23.6)	13,369 (24.1)		
None of the above	23,253 (15.2)	8,168 (14.7)		
A levels/AS levels or equivalent	18,949 (12.4)	6,921 (12.5)		
Other professional qualifications	8,951 (5.8)	3,143 (5.7)		
CSEs or equivalent	7,991 (5.2)	2,897 (5.2)		
NVQ/HND/HNC or equivalent	6,423 (4.2)	2,382 (4.3)		
**Townsend Deprivation Score***	-2.3 [-3.7, 0.1]	-2.3 [-3.7, 0.1]	0.003	KW
**Lifestyle score***	3.0 [2.0, 4.0]	3.0 [2.0, 4.0]	<0.001	KW
**BMI** (kg/m^2)	25.9 [23.3, 29.4]	25.8 [23.3, 29.3]	<0.001	KW
**APOE e4 alleles**, N (%)				
1 x e4 allele		50458 (91.0)		
2 x e4 allele		4965 (9.0)		

*Continuous data in median [Interquartile range] and categorical data as number (%).
Abbreviations: N = Number; O = Ordinary Level Qualification; GCSE = General Certificate of Secondary Education; A = Advanced Level Qualification; AS = Advanced Subsidiary Level Qualification; CSE = Certificate of Secondary Education; NVQ = National Vocational Qualification; HND = Higher National Diploma; HNC = Higher National Certificate; KW = Kruskal-Wallis.

Table S6| ICD-10 codes for autoimmune diseases, metabolic disorders and cancer.

Category	Disorder	ICD10 Code
Autoimmune disease*	Pernicious anemia	D51
Autoimmune disease	Autoimmune hemolytic anemia	D59.0, D59.1
Autoimmune disease	Paroxysmal nocturnal haemoglobinuria	D59.5
Autoimmune disease	Acquired pure red cell aplasia	D60
Autoimmune disease	Aplastic anaemia, unspecified	D61.9
Autoimmune disease	Haemorrhagic disorder due to circulating anticoagulants	D68.3
Autoimmune disease	Other Thrombophilia (e.g. Antiphospholipid syndrome)	D68.6
Autoimmune disease	Immune thrombocytopenic purpura	D69.2
Autoimmune disease	Idiopathic thrombocytopenic purpura	D69.3
Autoimmune disease	Other primary thrombocytopenia (e.g. Evans syndrome)	D69.4
Autoimmune disease	Sarcoidosis	D86.9
Autoimmune disease	Autoimmune disease, not elsewhere classified	D89.89
Autoimmune disease	Grave’s disease	E05.0
Autoimmune disease	Hashimoto’s thyroiditis	E06.3
Autoimmune disease	Type I diabetes	E10
Autoimmune disease	Addison disease	E27.2
Autoimmune disease	Autoimmune polyglandular failure	E31.0
Autoimmune disease	Other encephalitis, myelitis and encephalomyelitis	G04.8
Autoimmune disease	Multiple sclerosis	G35
Autoimmune disease	Neuromyelitis optica	G36.0
Autoimmune disease	Acute and subacute hemorrhagic leukoencephalitis	G36.1
Autoimmune disease	Concentric sclerosis	G37.5
Autoimmune disease	Neuralgic amyotrophy	G54.5
Autoimmune disease	Guillain–Barre´syndrome	G61.0
Autoimmune disease	Chronic inflammatory demyelinating polyneuritis	G61.81
Autoimmune disease	Myasthenia gravis	G70.0
Autoimmune disease	Inflammatory myopathy, not elsewhere classified	G72.4
Autoimmune disease	Acute rheumatic fever	I00-I02
Autoimmune disease	Myocarditis, unspecified	I51.4
Autoimmune disease	Raynaud’s syndrome	I73.0
Autoimmune disease	Chronic atrophic gastritis	K29.4
Autoimmune disease	Inflammatory bowel disease	K50-K52
Autoimmune disease	Primary biliary cirrhosis	K74.3
Autoimmune disease	Autoimmune hepatitis	K75.4
Autoimmune disease	Autoimmune pancreatitis	K86.1
Autoimmune disease	Celiac disease	K90.0
Autoimmune disease	Pemphigus	L10
Autoimmune disease	Bullous pemphigoid	L12.0
Autoimmune disease	Cicatricial pemphigoid	L12.1
Autoimmune disease	Dermatitis herpetiformis	L13.0
Autoimmune disease	Other specified bullous disorders	L13.8

Autoimmune disease	Autoimmune progesterone dermatitis	L30.8
Autoimmune disease	Psoriasis	L40
Autoimmune disease	Alopecia areata	L63
Autoimmune disease	Vitiligo	L80
Autoimmune disease	Discoid lupus erythematosus	L93.0
Autoimmune disease	Scleroderma	L94.0, L94.1
Autoimmune disease	Vasculitis limited to the skin, unspecified	L95.9
Autoimmune disease	Reactive arthritis	M02.9
Autoimmune disease	Rheumatoid arthritis	M06.9
Autoimmune disease	Juvenile rheumatoid arthritis	M08.0
Autoimmune disease	Palindromic rheumatism	M12.3
Autoimmune disease	Systemic connective tissue disorders	M30-M36
Autoimmune disease	Ankylosing spondylitis	M45.9
Autoimmune disease	Relapsing polychondritis	M94.1
Autoimmune disease	Salpingitis and oophoritis, unspecified	N70.9
Cancer	Malignant neoplasms	C00-C97
Metabolic disorders	Metabolic disorders	E70-E90

* Autoimmune diseases are selected based on Autoimmune disease list of the Global Autoimmune Institute (https://www.autoimmuneinstitute.org/resources/autoimmune-disease-list/)

Table S7| Associations between female-specific factors and late life cognition.

Model	Test	beta	S.E.	t	p	pFDR	d
Reproductive Span	PairsMatch	0.003	0.003	0.731	0.465	0.586	0.005
	ReactionTime	0.009	0.003	2.619	**0.009**	**0.025**	0.017
	SymbolSub	0.017	0.006	2.817	**0.005**	**0.016**	0.035
	DigitSpan	0.035	0.011	3.309	**0.001**	**0.003**	0.066
	TrailA	0.006	0.007	0.784	0.433	0.566	0.010
	TrailB	0.010	0.007	1.333	0.182	0.319	0.018
Age at Menarche	PairsMatch	0.003	0.002	1.271	0.204	0.336	0.006
	ReactionTime	-0.004	0.002	-1.675	0.094	0.191	-0.008
	SymbolSub	0.006	0.004	1.322	0.186	0.319	0.012
	DigitSpan	0.003	0.007	0.456	0.649	0.702	0.007
	TrailA	0.013	0.005	2.560	**0.010**	**0.028**	0.025
	TrailB	0.007	0.005	1.591	0.112	0.220	0.016
Age at Menopause	PairsMatch	0.005	0.003	1.339	0.181	0.319	0.009
	ReactionTime	0.008	0.003	2.346	**0.019**	**0.046**	0.015
	SymbolSub	0.019	0.006	3.062	**0.002**	**0.008**	0.037
	DigitSpan	0.037	0.010	3.564	**3.67e-04**	**0.001**	0.071
	TrailA	0.010	0.007	1.365	0.172	0.319	0.018
	TrailB	0.012	0.007	1.737	0.082	0.172	0.023
Oophorectomy	PairsMatch	0.009	0.009	0.941	0.347	0.487	0.009
	ReactionTime	-0.017	0.009	-1.846	0.065	0.141	-0.017
	SymbolSub	0.036	0.017	2.120	**0.034**	0.079	0.040
	DigitSpan	-0.038	0.029	-1.308	0.191	0.319	-0.037
	TrailA	0.023	0.020	1.178	0.239	0.371	0.024
	TrailB	0.012	0.019	0.622	0.534	0.618	0.013
Age at Oophorectomy	PairsMatch	-0.013	0.010	-1.241	0.215	0.346	-0.022
	ReactionTime	-0.006	0.010	-0.597	0.550	0.632	-0.011
	SymbolSub	-0.003	0.019	-0.164	0.870	0.897	-0.006
	DigitSpan	0.032	0.029	1.104	0.270	0.397	0.061
	TrailA	-0.028	0.023	-1.228	0.220	0.349	-0.049
	TrailB	-0.049	0.023	-2.161	**0.031**	0.073	-0.086
Hysterectomy	PairsMatch	-0.006	0.009	-0.716	0.474	0.590	-0.006
	ReactionTime	0.005	0.008	0.650	0.515	0.613	0.006
	SymbolSub	0.020	0.015	1.329	0.184	0.319	0.023
	DigitSpan	-0.071	0.027	-2.648	**0.008**	**0.025**	-0.072
	TrailA	-0.009	0.018	-0.502	0.616	0.683	-0.009
	TrailB	0.021	0.017	1.254	0.210	0.342	0.023
Age at Hysterectomy	PairsMatch	-0.011	0.008	-1.354	0.176	0.319	-0.022
	ReactionTime	0.004	0.008	0.513	0.608	0.680	0.008
	SymbolSub	-0.033	0.016	-2.085	**0.037**	0.084	-0.068
	DigitSpan	0.002	0.026	0.087	0.931	0.945	0.005
	TrailA	-0.025	0.019	-1.311	0.190	0.319	-0.046

	TrailB	-0.018	0.018	-0.993	0.321	0.465	-0.035
N Childbirth	PairsMatch	0.025	0.004	5.628	**1.82e-08**	**1.05e-07**	0.027
	ReactionTime	0.033	0.004	7.785	**6.99e-15**	**1.03e-13**	0.037
	SymbolSub	-0.029	0.010	-2.978	**0.003**	**0.010**	-0.027
	DigitSpan	-0.008	0.017	-0.474	0.636	0.693	-0.007
	TrailA	-0.046	0.011	-4.002	**6.29e-05**	**2.68e-04**	-0.039
	TrailB	0.006	0.011	0.555	0.579	0.655	0.005
N Childbirth^2	PairsMatch	-0.011	0.004	-2.533	**0.011**	**0.029**	-0.012
	ReactionTime	-0.033	0.004	-7.631	**2.34e-14**	**3.09e-13**	-0.036
	SymbolSub	-0.011	0.012	-0.888	0.374	0.515	-0.008
	DigitSpan	-0.013	0.019	-0.694	0.488	0.601	-0.010
	TrailA	-0.010	0.014	-0.665	0.506	0.613	-0.006
	TrailB	-0.028	0.014	-2.059	**0.039**	0.088	-0.020
Age last Birth	PairsMatch	0.011	0.003	3.780	**1.57e-04**	**0.001**	0.022
	ReactionTime	-0.002	0.003	-0.741	0.459	0.586	-0.004
	SymbolSub	0.002	0.005	0.350	0.726	0.779	0.004
	DigitSpan	0.024	0.009	2.618	**0.009**	**0.025**	0.047
	TrailA	0.016	0.006	2.642	**0.008**	**0.025**	0.032
	TrailB	0.014	0.006	2.431	**0.015**	**0.037**	0.030
Age first Birth	PairsMatch	0.023	0.003	7.443	**9.90e-14**	**1.01e-12**	0.043
	ReactionTime	0.020	0.003	6.727	**1.74e-11**	**1.35e-10**	0.039
	SymbolSub	0.032	0.005	6.001	**1.98e-09**	**1.31e-08**	0.068
	DigitSpan	0.049	0.009	5.209	**1.93e-07**	**9.44e-07**	0.094
	TrailA	0.045	0.006	7.202	**6.11e-13**	**5.76e-12**	0.088
	TrailB	0.045	0.006	7.593	**3.24e-14**	**3.56e-13**	0.093
Miscarriage & Termination	PairsMatch	-0.015	0.013	-1.102	0.271	0.397	-0.015
	ReactionTime	0.008	0.013	0.631	0.528	0.617	0.009
	SymbolSub	0.013	0.021	0.654	0.513	0.613	0.015
	DigitSpan	0.029	0.042	0.684	0.494	0.603	0.030
	TrailA	0.017	0.023	0.761	0.447	0.578	0.019
	TrailB	-0.012	0.022	-0.553	0.580	0.655	-0.014
Stillbirth	PairsMatch	0.004	0.077	0.058	0.954	0.961	0.005
	ReactionTime	-0.001	0.072	-0.009	0.993	0.993	-0.001
	SymbolSub	-0.018	0.131	-0.138	0.890	0.911	-0.021
	DigitSpan	-0.277	0.249	-1.111	0.266	0.397	-0.288
	TrailA	-0.254	0.152	-1.663	0.096	0.193	-0.274
	TrailB	-0.319	0.148	-2.161	**0.031**	0.073	-0.356
Current HT use	PairsMatch	-0.031	0.011	-2.911	**0.004**	**0.012**	-0.026
	ReactionTime	0.012	0.010	1.212	0.225	0.354	0.011
	SymbolSub	-0.013	0.017	-0.729	0.466	0.586	-0.012
	DigitSpan	0.032	0.034	0.947	0.344	0.487	0.016
	TrailA	0.040	0.020	1.993	**0.046**	0.102	0.022
	TrailB	-0.029	0.019	-1.501	0.133	0.255	-0.017

Past HT use	PairsMatch	-0.033	0.006	-5.394	**6.89e-08**	**3.64e-07**	-0.048
	ReactionTime	0.023	0.006	3.911	**9.19e-05**	**3.79e-04**	0.036
	SymbolSub	-0.029	0.011	-2.763	**0.006**	**0.018**	-0.046
	DigitSpan	0.005	0.019	0.242	0.809	0.854	0.004
	TrailA	0.010	0.013	0.826	0.409	0.550	0.009
	TrailB	-0.051	0.012	-4.299	**1.72e-05**	**7.56e-05**	-0.048
Age HT initiation	PairsMatch	-0.002	0.005	-0.478	0.633	0.693	-0.004
	ReactionTime	0.007	0.004	1.546	0.122	0.237	0.013
	SymbolSub	0.021	0.008	2.588	**0.010**	**0.027**	0.042
	DigitSpan	0.015	0.014	1.105	0.269	0.397	0.029
	TrailA	0.003	0.010	0.323	0.747	0.795	0.006
	TrailB	0.009	0.009	0.982	0.326	0.468	0.017
Age HT rel. Menopause	PairsMatch	-0.004	0.005	-0.815	0.415	0.554	-0.008
	ReactionTime	0.001	0.005	0.188	0.851	0.885	0.002
	SymbolSub	0.008	0.009	0.802	0.422	0.558	0.016
	DigitSpan	-0.003	0.016	-0.191	0.848	0.885	-0.006
	TrailA	0.015	0.012	1.323	0.186	0.319	0.028
	TrailB	0.029	0.011	2.562	**0.010**	**0.028**	0.054
Duration HT use	PairsMatch	-0.004	0.005	-0.836	0.403	0.548	-0.007
	ReactionTime	0.005	0.004	1.112	0.266	0.397	0.009
	SymbolSub	-0.015	0.008	-1.732	0.083	0.172	-0.028
	DigitSpan	0.009	0.014	0.631	0.528	0.617	0.017
	TrailA	0.009	0.010	0.915	0.360	0.501	0.016
	TrailB	-0.017	0.009	-1.751	0.080	0.170	-0.031
Current HC use	PairsMatch	0.046	0.018	2.558	**0.011**	**0.028**	0.021
	ReactionTime	0.145	0.017	8.440	**3.19e-17**	**8.41e-16**	0.059
	SymbolSub	0.154	0.029	5.387	**7.19e-08**	**3.65e-07**	0.069
	DigitSpan	0.198	0.054	3.666	**2.47e-04**	**0.001**	0.072
	TrailA	0.158	0.033	4.836	**1.33e-06**	**6.07e-06**	0.068
	TrailB	0.190	0.031	6.085	**1.18e-09**	**8.64e-09**	0.087
Past HC use	PairsMatch	0.016	0.006	2.521	**0.012**	**0.030**	0.020
	ReactionTime	0.091	0.006	14.795	**1.70e-49**	**2.25e-47**	0.113
	SymbolSub	0.105	0.012	9.023	**1.89e-19**	**6.25e-18**	0.116
	DigitSpan	0.116	0.020	5.688	**1.30e-08**	**7.83e-08**	0.113
	TrailA	0.094	0.014	6.763	**1.37e-11**	**1.13e-10**	0.089
	TrailB	0.109	0.013	8.239	**1.79e-16**	**3.93e-15**	0.117
Age HC initiation	PairsMatch	-0.015	0.003	-5.195	**2.05e-07**	**9.65e-07**	-0.028
	ReactionTime	-0.028	0.003	-9.927	**3.22e-23**	**1.42e-21**	-0.053
	SymbolSub	-0.050	0.005	-10.117	**4.94e-24**	**3.26e-22**	-0.100
	DigitSpan	-0.071	0.009	-7.603	**3.08e-14**	**3.56e-13**	-0.126
	TrailA	-0.033	0.006	-5.699	**1.21e-08**	**7.63e-08**	-0.060
	TrailB	-0.044	0.006	-8.014	**1.15e-15**	**1.89e-14**	-0.085
Duration HC use	PairsMatch	0.004	0.003	1.385	0.166	0.313	0.008

	ReactionTime	0.008	0.003	2.934	**0.003**	**0.011**	0.016
	SymbolSub	0.036	0.004	8.057	**8.06e-16**	**1.52e-14**	0.082
	DigitSpan	0.047	0.008	5.596	**2.23e-08**	**1.23e-07**	0.095
	TrailA	0.035	0.005	6.908	**4.99e-12**	**4.39e-11**	0.075
	TrailB	0.030	0.005	6.045	**1.51e-09**	**1.05e-08**	0.066

Abbreviation: PairMatch = Pair Matching Test, ReactionTime = Reaction Time Test, SymbolSub = Symbol Substitution Test, DigitSpan = Digit Span Test, Trail A & B = Trail Making Test A & B, N = Number, HT = Hormone Therapy, HC = Hormonal Contraceptive, S.E. = Standard Error, FDR = False Discovery Rate. Significant results are highlighted in bold.

Table S8| Non-linear effects of number of childbirths on cognitive functioning.

	**Reaction Time**				**Pair Matching**			
**N Childbirth**	**beta**	**S.E.**	**t**	**p**	**beta**	**S.E.**	**t**	**p**
0 (intercept)	-0.036	0.005	-7.032	**2.05e-12**	-0.032	0.005	-5.848	**4.99e-09**
1	0.035	0.008	4.362	**1.29e-05**	0.021	0.008	2.498	**0.0125**
2	0.059	0.006	9.444	**< 2e-16**	0.031	0.007	4.767	**1.87e-06**
3	0.042	0.008	5.580	**2.40e-08**	0.051	0.008	6.524	**6.87e-11**
4	-0.002	0.012	-0.143	0.886	0.054	0.013	4.317	**1.58e-05**
5	-0.066	0.024	-2.719	**0.007**	0.025	0.026	0.963	0.336
6	-0.107	0.046	-2.341	**0.019**	0.022	0.047	0.467	0.640
7+	-0.107	0.063	1.715	0.086	0.061	0.065	0.950	0.342

Abbreviation: N = Number, S.E. = Standard Error

Table S9| Associations between female-specific factors and late life cognition after removal of extreme values.

**Model**	**Test**	**beta**	**S.E.**	**t**	**p**	**p_{FDR}**	**d**
Reproductive Span	PairsMatch	0.004	0.003	1.150	0.250	0.393	0.007
	ReactionTime	0.008	0.003	2.455	**0.014**	**0.038**	0.016
	SymbolSub	0.015	0.006	2.436	**0.015**	**0.039**	0.030
	DigitSpan	0.037	0.010	3.538	**4.05e-04**	**0.002**	0.072
	TrailA	0.008	0.007	1.077	0.281	0.413	0.014
	TrailB	0.010	0.007	1.416	0.157	0.291	0.019
Age at Menarche	PairsMatch	0.003	0.002	1.364	0.173	0.312	0.007
	ReactionTime	-0.001	0.002	-0.496	0.620	0.682	-0.002
	SymbolSub	0.007	0.004	1.598	0.110	0.220	0.015
	DigitSpan	0.008	0.007	1.032	0.302	0.424	0.016
	TrailA	0.013	0.005	2.614	**0.009**	**0.028**	0.026
	TrailB	0.008	0.005	1.710	0.087	0.186	0.017
Age at Menopause	PairsMatch	0.005	0.003	1.570	0.117	0.230	0.010
	ReactionTime	0.008	0.003	2.549	**0.011**	**0.032**	0.016
	SymbolSub	0.017	0.006	2.763	**0.006**	**0.019**	0.034
	DigitSpan	0.041	0.010	3.955	**7.70e-05**	**3.18e-04**	0.079
	TrailA	0.012	0.007	1.722	0.085	0.184	0.023
	TrailB	0.012	0.007	1.701	0.089	0.186	0.023
Oophorectomy	PairsMatch	0.009	0.009	0.941	0.347	0.477	0.009
	ReactionTime	-0.017	0.009	-1.846	0.065	0.148	-0.017
	SymbolSub	0.036	0.017	2.120	**0.034**	0.085	0.040
	DigitSpan	-0.038	0.029	-1.308	0.191	0.327	-0.037
	TrailA	0.023	0.020	1.178	0.239	0.379	0.024
	TrailB	0.012	0.019	0.622	0.534	0.623	0.013
Age at Oophorectomy	PairsMatch	-0.011	0.010	-1.106	0.269	0.406	-0.020
	ReactionTime	-0.008	0.010	-0.765	0.444	0.574	-0.014
	SymbolSub	0.000	0.019	0.025	0.980	0.987	0.001
	DigitSpan	0.031	0.029	1.061	0.289	0.414	0.059
	TrailA	-0.018	0.023	-0.771	0.441	0.574	-0.031
	TrailB	-0.040	0.023	-1.772	0.076	0.168	-0.070
Hysterectomy	PairsMatch	-0.006	0.009	-0.716	0.474	0.582	-0.006
	ReactionTime	0.005	0.008	0.650	0.515	0.613	0.006
	SymbolSub	0.020	0.015	1.329	0.184	0.327	0.023
	DigitSpan	-0.071	0.027	-2.648	**0.008**	**0.026**	-0.072
	TrailA	-0.009	0.018	-0.502	0.616	0.682	-0.009
	TrailB	0.021	0.017	1.254	0.210	0.355	0.023
Age at Hysterectomy	PairsMatch	-0.010	0.008	-1.234	0.217	0.363	-0.020
	ReactionTime	0.003	0.008	0.336	0.737	0.791	0.005
	SymbolSub	-0.029	0.015	-1.893	0.058	0.135	-0.062

	DigitSpan	0.012	0.027	0.459	0.647	0.700	0.024
	TrailA	-0.025	0.019	-1.323	0.186	0.327	-0.047
	TrailB	-0.020	0.018	-1.090	0.276	0.409	-0.038
N Childbirth	PairsMatch	0.026	0.006	4.423	**9.74e-06**	**4.43e-05**	0.021
	ReactionTime	0.064	0.006	11.277	**1.75e-29**	**1.15e-27**	0.054
	SymbolSub	-0.023	0.010	-2.243	**0.025**	0.064	-0.020
	DigitSpan	-0.013	0.018	-0.713	0.476	0.582	-0.011
	TrailA	-0.040	0.012	-3.397	**0.001**	**0.003**	-0.033
	TrailB	0.007	0.011	0.615	0.538	0.623	0.006
N Childbirth^2	PairsMatch	-0.012	0.006	-1.955	0.051	0.119	-0.009
	ReactionTime	-0.063	0.006	-11.023	**3.04e-28**	**1.34e-26**	-0.053
	SymbolSub	-0.017	0.011	-1.502	0.133	0.252	-0.014
	DigitSpan	-0.005	0.018	-0.252	0.801	0.852	-0.004
	TrailA	-0.014	0.013	-1.070	0.285	0.413	-0.010
	TrailB	-0.025	0.012	-1.990	**0.047**	0.112	-0.019
Age last Birth	PairsMatch	0.012	0.003	4.156	**3.24e-05**	**1.38e-04**	0.024
	ReactionTime	-0.003	0.003	-0.872	0.383	0.521	-0.005
	SymbolSub	0.002	0.005	0.458	0.647	0.700	0.005
	DigitSpan	0.022	0.009	2.451	**0.014**	**0.038**	0.044
	TrailA	0.016	0.006	2.606	**0.009**	**0.028**	0.032
	TrailB	0.016	0.006	2.753	**0.006**	**0.019**	0.034
Age first Birth	PairsMatch	0.022	0.003	7.191	**6.48e-13**	**6.58e-12**	0.042
	ReactionTime	0.018	0.003	6.070	**1.28e-09**	**9.41e-09**	0.036
	SymbolSub	0.032	0.005	5.982	**2.23e-09**	**1.47e-08**	0.068
	DigitSpan	0.049	0.010	5.204	**1.99e-07**	**1.05e-06**	0.094
	TrailA	0.043	0.006	6.914	**4.83e-12**	**4.56e-11**	0.085
	TrailB	0.046	0.006	7.744	**9.96e-15**	**1.31e-13**	0.095
Miscarriage & Termination	PairsMatch	-0.015	0.013	-1.102	0.271	0.406	-0.015
	ReactionTime	0.008	0.013	0.631	0.528	0.623	0.009
	SymbolSub	0.013	0.021	0.654	0.513	0.613	0.015
	DigitSpan	0.029	0.042	0.684	0.494	0.598	0.030
	TrailA	0.017	0.023	0.761	0.447	0.574	0.019
	TrailB	-0.012	0.022	-0.553	0.580	0.650	-0.014
Stillbirth	PairsMatch	0.004	0.077	0.058	0.954	0.968	0.005
	ReactionTime	-0.001	0.072	-0.009	0.993	0.993	-0.001
	SymbolSub	-0.018	0.131	-0.138	0.890	0.918	-0.021
	DigitSpan	-0.277	0.249	-1.111	0.266	0.406	-0.288
	TrailA	-0.254	0.152	-1.663	0.096	0.199	-0.274
	TrailB	-0.319	0.148	-2.161	**0.031**	0.078	-0.356
Current HT use	PairsMatch	-0.031	0.011	-2.911	**0.004**	**0.013**	-0.026
	ReactionTime	0.012	0.010	1.212	0.225	0.368	0.011
	SymbolSub	-0.013	0.017	-0.729	0.466	0.580	-0.012
	DigitSpan	0.032	0.034	0.947	0.344	0.477	0.016

	TrailA	0.040	0.020	1.993	**0.046**	0.112	0.022
	TrailB	-0.029	0.019	-1.501	0.133	0.252	-0.017
Past HT use	PairsMatch	-0.033	0.006	-5.394	**6.89e-08**	**3.95e-07**	-0.048
	ReactionTime	0.023	0.006	3.911	**9.19e-05**	**3.68e-04**	0.036
	SymbolSub	-0.029	0.011	-2.763	**0.006**	**0.019**	-0.046
	DigitSpan	0.005	0.019	0.242	0.809	0.854	0.004
	TrailA	0.010	0.013	0.826	0.409	0.550	0.009
	TrailB	-0.051	0.012	-4.299	**1.72e-05**	**7.56e-05**	-0.048
Age HT initiation	PairsMatch	0.000	0.005	-0.088	0.930	0.951	-0.001
	ReactionTime	0.006	0.004	1.312	0.190	0.327	0.011
	SymbolSub	0.012	0.008	1.500	0.134	0.252	0.025
	DigitSpan	0.010	0.014	0.745	0.456	0.574	0.020
	TrailA	0.005	0.010	0.552	0.581	0.650	0.010
	TrailB	0.007	0.009	0.756	0.450	0.574	0.013
Age HT rel. Menopause	PairsMatch	-0.003	0.005	-0.586	0.558	0.635	-0.006
	ReactionTime	-0.001	0.005	-0.225	0.822	0.861	-0.002
	SymbolSub	0.007	0.009	0.791	0.429	0.572	0.016
	DigitSpan	-0.003	0.016	-0.197	0.844	0.877	-0.006
	TrailA	0.012	0.011	1.041	0.298	0.423	0.022
	TrailB	0.027	0.011	2.501	**0.012**	**0.035**	0.053
Duration HT use	PairsMatch	-0.005	0.005	-1.190	0.234	0.377	-0.010
	ReactionTime	0.005	0.005	1.109	0.267	0.406	0.009
	SymbolSub	-0.012	0.009	-1.404	0.160	0.294	-0.023
	DigitSpan	0.010	0.014	0.744	0.457	0.574	0.020
	TrailA	0.006	0.010	0.595	0.552	0.633	0.011
	TrailB	-0.016	0.010	-1.644	0.100	0.203	-0.030
Current HC use	PairsMatch	0.046	0.018	2.558	**0.011**	**0.032**	0.021
	ReactionTime	0.145	0.017	8.440	**3.19e-17**	**6.01e-16**	0.059
	SymbolSub	0.154	0.029	5.387	**7.19e-08**	**3.95e-07**	0.069
	DigitSpan	0.198	0.054	3.666	**2.47e-04**	**0.001**	0.072
	TrailA	0.158	0.033	4.836	**1.33e-06**	**6.28e-06**	0.068
	TrailB	0.190	0.031	6.085	**1.18e-09**	**9.15e-09**	0.087
Past HC use	PairsMatch	0.016	0.006	2.521	**0.012**	**0.034**	0.020
	ReactionTime	0.091	0.006	14.795	**1.70e-49**	**2.25e-47**	0.113
	SymbolSub	0.105	0.012	9.023	**1.89e-19**	**4.17e-18**	0.116
	DigitSpan	0.116	0.020	5.688	**1.30e-08**	**7.83e-08**	0.113
	TrailA	0.094	0.014	6.763	**1.37e-11**	**1.21e-10**	0.089
	TrailB	0.109	0.013	8.239	**1.79e-16**	**2.95e-15**	0.117
Age HC initiation	PairsMatch	-0.018	0.003	-5.970	**2.37e-09**	**1.49e-08**	-0.032
	ReactionTime	-0.027	0.003	-9.142	**6.21e-20**	**1.64e-18**	-0.049
	SymbolSub	-0.050	0.005	-9.676	**4.04e-22**	**1.33e-20**	-0.096
	DigitSpan	-0.073	0.010	-7.395	**1.49e-13**	**1.79e-12**	-0.123
	TrailA	-0.030	0.006	-4.961	**7.04e-07**	**3.44e-06**	-0.053

	TrailB	-0.042	0.006	-7.335	**2.26e-13**	**2.49e-12**	-0.078
Duration HC use	PairsMatch	0.003	0.003	1.211	0.226	0.368	0.007
	ReactionTime	0.005	0.003	1.786	0.074	0.166	0.010
	SymbolSub	0.036	0.005	7.866	**3.76e-15**	**5.51e-14**	0.081
	DigitSpan	0.044	0.009	5.168	**2.40e-07**	**1.22e-06**	0.089
	TrailA	0.034	0.005	6.601	**4.14e-11**	**3.42e-10**	0.072
	TrailB	0.030	0.005	6.057	**1.40e-09**	**9.72e-09**	0.066

Abbreviation: PairMatch = Pair Matching Test. ReactionTime = Reaction Time Test. SymbolSub = Symbol Substitution Test. DigitSpan = Digit Span Test. Trail A & B = Trail Making Test A & B. N = Number. HT = Hormone Therapy. HC = Hormonal Contraceptive. S.E. = Standard Error. FDR = False Discovery Rate. Significant results are highlighted in bold.

Table S10| Associations between female-specific factors and late life cognition, including participants with ICD-10 diagnoses known to impact the brain and cognition.

**Model**	**Test**	**beta**	**S.E.**	**t**	**p**	**p_{FDR}**	**d**
Reproductive Span	PairsMatch	0.001	0.003	0.370	0.712	0.794	0.002
	ReactionTime	0.014	0.003	4.368	**1.25e-05**	**5.01e-05**	0.026
	SymbolSub	0.021	0.006	3.545	**3.94e-04**	**0.001**	0.042
	DigitSpan	0.044	0.010	4.524	**6.13e-06**	**2.53e-05**	0.084
	TrailA	0.011	0.007	1.552	0.121	0.213	0.020
	TrailB	0.017	0.007	2.432	**0.015**	**0.036**	0.031
Age at Menarche	PairsMatch	0.003	0.002	1.279	0.201	0.320	0.006
	ReactionTime	-0.005	0.002	-2.120	**0.034**	0.072	-0.010
	SymbolSub	0.005	0.004	1.236	0.216	0.332	0.011
	DigitSpan	0.005	0.007	0.730	0.465	0.574	0.010
	TrailA	0.013	0.005	2.761	**0.006**	**0.014**	0.026
	TrailB	0.009	0.004	2.114	**0.035**	0.072	0.020
Age at Menopause	PairsMatch	0.003	0.003	0.928	0.353	0.471	0.005
	ReactionTime	0.013	0.003	4.132	**3.59e-05**	**1.40e-04**	0.025
	SymbolSub	0.023	0.006	3.778	**1.59e-04**	**0.001**	0.044
	DigitSpan	0.048	0.010	4.938	**8.00e-07**	**3.52e-06**	0.091
	TrailA	0.016	0.007	2.263	**0.024**	0.054	0.028
	TrailB	0.022	0.007	3.148	**0.002**	**0.005**	0.040
Oophorectomy	PairsMatch	0.007	0.009	0.836	0.403	0.514	0.007
	ReactionTime	-0.018	0.008	-2.183	**0.029**	0.065	-0.018
	SymbolSub	0.029	0.016	1.864	0.062	0.118	0.032
	DigitSpan	-0.052	0.026	-2.000	**0.046**	0.090	-0.051
	TrailA	0.027	0.018	1.469	0.142	0.237	0.028
	TrailB	0.014	0.018	0.769	0.442	0.550	0.014
Age at Oophorectomy	PairsMatch	-0.014	0.009	-1.525	0.127	0.221	-0.025
	ReactionTime	0.005	0.009	0.537	0.591	0.697	0.009
	SymbolSub	-0.005	0.018	-0.274	0.784	0.835	-0.009
	DigitSpan	0.044	0.026	1.664	0.096	0.174	0.082
	TrailA	-0.027	0.022	-1.263	0.207	0.325	-0.046
	TrailB	-0.039	0.021	-1.864	0.062	0.118	-0.068
Hysterectomy	PairsMatch	-0.001	0.008	-0.151	0.880	0.915	-0.001
	ReactionTime	0.005	0.007	0.659	0.510	0.617	0.005
	SymbolSub	0.015	0.014	1.079	0.281	0.399	0.017
	DigitSpan	-0.072	0.024	-2.995	**0.003**	**0.008**	-0.073
	TrailA	-0.014	0.017	-0.833	0.405	0.514	-0.014
	TrailB	0.007	0.016	0.459	0.646	0.742	0.008
Age at Hysterectomy	PairsMatch	-0.015	0.008	-1.917	0.055	0.107	-0.028
	ReactionTime	0.008	0.007	1.153	0.249	0.361	0.017
	SymbolSub	-0.022	0.015	-1.509	0.131	0.225	-0.045
	DigitSpan	0.008	0.024	0.348	0.728	0.800	0.016
	TrailA	-0.015	0.018	-0.878	0.380	0.502	-0.029
	TrailB	-0.004	0.017	-0.210	0.834	0.881	-0.007
N Childbirth	PairsMatch	0.023	0.004	5.607	**2.07e-08**	**1.05e-07**	0.025
	ReactionTime	0.038	0.004	9.282	**1.68e-20**	**4.44e-19**	0.041
	SymbolSub	-0.027	0.009	-3.006	**0.003**	**0.008**	-0.026

	DigitSpan	-0.019	0.016	-1.171	0.242	0.359	-0.016
	TrailA	-0.043	0.011	-4.119	**3.81e-05**	**1.44e-04**	-0.038
	TrailB	0.009	0.010	0.848	0.396	0.514	0.008
N Childbirth[2]	PairsMatch	-0.010	0.004	-2.451	**0.014**	**0.035**	-0.011
	ReactionTime	-0.037	0.004	-9.037	**1.62e-19**	**3.57e-18**	-0.040
	SymbolSub	-0.014	0.011	-1.281	0.200	0.320	-0.011
	DigitSpan	0.001	0.018	0.068	0.946	0.960	0.001
	TrailA	-0.011	0.013	-0.841	0.400	0.514	-0.008
	TrailB	-0.030	0.012	-2.367	**0.018**	**0.042**	-0.022
Age last Birth	PairsMatch	0.009	0.003	3.084	**0.002**	**0.006**	0.017
	ReactionTime	-0.001	0.003	-0.316	0.752	0.814	-0.002
	SymbolSub	0.002	0.005	0.429	0.668	0.753	0.005
	DigitSpan	0.025	0.008	2.936	**0.003**	**0.009**	0.049
	TrailA	0.017	0.006	2.906	**0.004**	**0.010**	0.034
	TrailB	0.018	0.006	3.234	**0.001**	**0.004**	0.038
Age first Birth	PairsMatch	0.022	0.003	7.484	**7.23e-14**	**6.36e-13**	0.041
	ReactionTime	0.022	0.003	8.052	**8.20e-16**	**1.08e-14**	0.044
	SymbolSub	0.033	0.005	6.398	**1.60e-10**	**1.24e-09**	0.069
	DigitSpan	0.050	0.009	5.734	**1.00e-08**	**5.30e-08**	0.096
	TrailA	0.046	0.006	7.650	**2.07e-14**	**1.96e-13**	0.089
	TrailB	0.049	0.006	8.622	**6.90e-18**	**1.01e-16**	0.101
Miscarriage & Termination	PairsMatch	-0.014	0.013	-1.137	0.256	0.367	-0.014
	ReactionTime	0.015	0.012	1.291	0.197	0.320	0.016
	SymbolSub	0.012	0.019	0.639	0.523	0.627	0.014
	DigitSpan	0.031	0.038	0.814	0.416	0.523	0.033
	TrailA	0.008	0.022	0.364	0.716	0.794	0.009
	TrailB	-0.021	0.021	-0.979	0.328	0.446	-0.023
Stillbirth	PairsMatch	-0.004	0.069	-0.058	0.954	0.961	-0.004
	ReactionTime	-0.012	0.066	-0.175	0.861	0.902	-0.012
	SymbolSub	-0.005	0.124	-0.037	0.970	0.970	-0.005
	DigitSpan	-0.216	0.226	-0.953	0.341	0.459	-0.226
	TrailA	-0.247	0.144	-1.714	0.087	0.159	-0.265
	TrailB	-0.399	0.139	-2.862	**0.004**	**0.011**	-0.443
Current HT use	PairsMatch	-0.032	0.010	-3.335	**0.001**	**0.003**	-0.027
	ReactionTime	0.013	0.009	1.404	0.160	0.265	0.011
	SymbolSub	-0.008	0.016	-0.513	0.608	0.704	-0.008
	DigitSpan	0.038	0.031	1.238	0.216	0.332	0.019
	TrailA	0.038	0.019	2.004	**0.045**	0.090	0.021
	TrailB	-0.030	0.018	-1.656	0.098	0.174	-0.017
Past HT use	PairsMatch	-0.034	0.006	-5.885	**4.00e-09**	**2.29e-08**	-0.047
	ReactionTime	0.020	0.005	3.707	**2.10e-04**	**0.001**	0.031
	SymbolSub	-0.032	0.010	-3.166	**0.002**	**0.005**	-0.049
	DigitSpan	0.005	0.018	0.303	0.762	0.817	0.005
	TrailA	0.012	0.012	0.995	0.320	0.443	0.010
	TrailB	-0.051	0.011	-4.534	**5.79e-06**	**2.47e-05**	-0.048
Age HT initiation	PairsMatch	-4.23e-04	0.004	-0.101	0.920	0.941	-0.001
	ReactionTime	0.009	0.004	2.129	**0.033**	0.072	0.016
	SymbolSub	0.021	0.008	2.817	**0.005**	**0.013**	0.043
	DigitSpan	0.028	0.013	2.163	**0.031**	0.067	0.052

	TrailA	0.004	0.009	0.445	0.656	0.746	0.007
	TrailB	0.016	0.009	1.764	0.078	0.145	0.029
Age HT rel. Menopause	PairsMatch	-0.001	0.005	-0.134	0.893	0.921	-0.001
	ReactionTime	-0.003	0.005	-0.691	0.489	0.598	-0.006
	SymbolSub	0.005	0.009	0.520	0.603	0.704	0.009
	DigitSpan	-0.016	0.015	-1.039	0.299	0.419	-0.030
	TrailA	0.004	0.011	0.339	0.735	0.801	0.007
	TrailB	0.021	0.011	2.018	**0.044**	0.089	0.040
Duration HT use	PairsMatch	-0.004	0.004	-0.990	0.322	0.443	-0.008
	ReactionTime	0.005	0.004	1.152	0.249	0.361	0.009
	SymbolSub	-0.009	0.008	-1.175	0.240	0.359	-0.018
	DigitSpan	0.007	0.013	0.590	0.555	0.660	0.014
	TrailA	0.011	0.009	1.193	0.233	0.353	0.020
	TrailB	-0.018	0.009	-2.046	**0.041**	0.084	-0.034
Current HC use	PairsMatch	0.047	0.017	2.769	**0.006**	**0.014**	0.021
	ReactionTime	0.152	0.016	9.328	**1.09e-20**	**3.59e-19**	0.062
	SymbolSub	0.150	0.028	5.462	**4.73e-08**	**2.23e-07**	0.067
	DigitSpan	0.185	0.051	3.628	**2.86e-04**	**0.001**	0.066
	TrailA	0.158	0.031	5.053	**4.36e-07**	**1.98e-06**	0.067
	TrailB	0.173	0.030	5.775	**7.74e-09**	**4.25e-08**	0.078
Past HC use	PairsMatch	0.014	0.006	2.349	**0.019**	**0.044**	0.018
	ReactionTime	0.092	0.006	16.023	**9.64e-58**	**1.27e-55**	0.116
	SymbolSub	0.100	0.011	9.009	**2.16e-19**	**4.07e-18**	0.111
	DigitSpan	0.111	0.019	5.887	**3.99e-09**	**2.29e-08**	0.107
	TrailA	0.083	0.013	6.338	**2.35e-10**	**1.73e-09**	0.079
	TrailB	0.098	0.013	7.764	**8.39e-15**	**8.52e-14**	0.105
Age HC initiation	PairsMatch	-0.017	0.003	-6.007	**1.89e-09**	**1.19e-08**	-0.030
	ReactionTime	-0.028	0.003	-10.586	**3.51e-26**	**1.55e-24**	-0.053
	SymbolSub	-0.052	0.005	-10.966	**6.07e-28**	**4.01e-26**	-0.103
	DigitSpan	-0.062	0.009	-7.153	**8.82e-13**	**7.28e-12**	-0.109
	TrailA	-0.035	0.006	-6.217	**5.11e-10**	**3.37e-09**	-0.063
	TrailB	-0.042	0.005	-7.841	**4.60e-15**	**5.52e-14**	-0.079
Duration HC use	PairsMatch	0.004	0.003	1.470	0.141	0.237	0.008
	ReactionTime	0.008	0.002	3.424	**0.001**	**0.002**	0.018
	SymbolSub	0.038	0.004	8.900	**5.81e-19**	**9.59e-18**	0.086
	DigitSpan	0.043	0.008	5.482	**4.26e-08**	**2.08e-07**	0.087
	TrailA	0.038	0.005	7.803	**6.18e-15**	**6.80e-14**	0.081
	TrailB	0.029	0.005	6.227	**4.80e-10**	**3.33e-09**	0.064

Abbreviation: PairMatch = Pair Matching Test. ReactionTime = Reaction Time Test. SymbolSub = Symbol Substitution Test. DigitSpan = Digit Span Test. Trail A & B = Trail Making Test A & B. N = Number. HT = Hormone Therapy. HC = Hormonal Contraceptive. S.E. = Standard Error. FDR = False Discovery Rate. Significant results are highlighted in bold.

Table S11| Associations between female-specific factors and late life cognition after adjustment for APOE ε4 genotype.

**Model**	**Test**	**beta**	**S.E.**	**t**	**p**	**p_{FDR}**	**d**
Reproductive Span	PairsMatch	0.002	0.004	0.662	0.508	0.612	0.004
	ReactionTime	0.008	0.003	2.444	**0.015**	**0.038**	0.016
	SymbolSub	0.015	0.006	2.414	**0.016**	**0.041**	0.030
	DigitSpan	0.037	0.011	3.404	**0.001**	**0.002**	0.070
	TrailA	0.006	0.008	0.836	0.403	0.537	0.011
	TrailB	0.008	0.007	1.080	0.280	0.425	0.015
Age at Menarche	PairsMatch	0.002	0.002	0.934	0.350	0.503	0.005
	ReactionTime	-0.004	0.002	-1.680	0.093	0.195	-0.008
	SymbolSub	0.006	0.004	1.335	0.182	0.324	0.013
	DigitSpan	0.004	0.008	0.524	0.600	0.666	0.008
	TrailA	0.013	0.005	2.521	**0.012**	**0.032**	0.025
	TrailB	0.007	0.005	1.490	0.136	0.264	0.015
Age at Menopause	PairsMatch	0.004	0.004	1.072	0.284	0.426	0.007
	ReactionTime	0.007	0.003	2.029	**0.043**	0.098	0.013
	SymbolSub	0.017	0.006	2.640	**0.008**	**0.024**	0.033
	DigitSpan	0.040	0.011	3.744	**1.82e-04**	**0.001**	0.076
	TrailA	0.011	0.008	1.398	0.162	0.301	0.019
	TrailB	0.011	0.007	1.468	0.142	0.272	0.020
Oophorectomy	PairsMatch	0.011	0.010	1.147	0.251	0.400	0.011
	ReactionTime	-0.021	0.009	-2.301	**0.021**	0.051	-0.022
	SymbolSub	0.041	0.017	2.369	**0.018**	**0.045**	0.045
	DigitSpan	-0.039	0.030	-1.328	0.184	0.324	-0.039
	TrailA	0.031	0.020	1.498	0.134	0.264	0.031
	TrailB	0.017	0.020	0.881	0.378	0.515	0.018
Age at Oophorectomy	PairsMatch	-0.017	0.010	-1.638	0.101	0.209	-0.030
	ReactionTime	-0.006	0.010	-0.624	0.533	0.622	-0.012
	SymbolSub	-0.005	0.020	-0.278	0.781	0.825	-0.010
	DigitSpan	0.038	0.030	1.270	0.204	0.339	0.072
	TrailA	-0.030	0.024	-1.266	0.206	0.339	-0.051
	TrailB	-0.049	0.023	-2.128	**0.033**	0.079	-0.087
Hysterectomy	PairsMatch	-0.004	0.009	-0.487	0.626	0.683	-0.002
	ReactionTime	0.003	0.008	0.384	0.701	0.752	0.003
	SymbolSub	0.020	0.015	1.290	0.197	0.339	0.022
	DigitSpan	-0.071	0.028	-2.571	**0.010**	**0.029**	-0.071
	TrailA	0.002	0.018	0.130	0.897	0.932	0.002
	TrailB	0.026	0.017	1.512	0.130	0.264	0.028
Age at Hysterectomy	PairsMatch	-0.011	0.009	-1.230	0.219	0.357	-0.020
	ReactionTime	0.006	0.008	0.674	0.501	0.612	0.011
	SymbolSub	-0.031	0.016	-1.959	**0.050**	0.110	-0.065

	DigitSpan	0.002	0.027	0.087	0.931	0.952	0.005
	TrailA	-0.022	0.019	-1.132	0.258	0.404	-0.041
	TrailB	-0.017	0.018	-0.909	0.364	0.511	-0.033
N Childbirth	PairsMatch	0.025	0.004	5.701	**1.19e-08**	**7.73e-08**	0.028
	ReactionTime	0.032	0.004	7.434	**1.06e-13**	**1.55e-12**	0.036
	SymbolSub	-0.031	0.010	-3.052	**0.002**	**0.008**	-0.028
	DigitSpan	-0.009	0.018	-0.528	0.598	0.666	-0.008
	TrailA	-0.046	0.012	-3.975	**7.06e-05**	**2.91e-04**	-0.040
	TrailB	0.008	0.011	0.711	0.477	0.597	0.007
N Childbirth^2	PairsMatch	-0.012	0.004	-2.712	**0.007**	**0.021**	-0.013
	ReactionTime	-0.031	0.004	-7.098	**1.27e-12**	1.19e-11	-0.035
	SymbolSub	-0.011	0.013	-0.864	0.387	0.522	-0.008
	DigitSpan	-0.010	0.020	-0.530	0.596	0.666	-0.008
	TrailA	-0.011	0.015	-0.725	0.468	0.595	-0.007
	TrailB	-0.033	0.014	-2.324	**0.020**	**0.049**	-0.023
Age last Birth	PairsMatch	0.011	0.003	3.444	**0.001**	**0.002**	0.021
	ReactionTime	-0.002	0.003	-0.796	0.426	0.557	-0.005
	SymbolSub	0.000	0.005	0.069	0.945	0.960	0.001
	DigitSpan	0.027	0.009	2.916	**0.004**	**0.012**	0.054
	TrailA	0.016	0.006	2.514	**0.012**	**0.032**	0.032
	TrailB	0.012	0.006	2.000	**0.046**	0.102	0.025
Age first Birth	PairsMatch	0.023	0.003	7.173	**7.39e-13**	**8.06e-12**	0.043
	ReactionTime	0.020	0.003	6.545	**5.97e-11**	**4.92e-10**	0.039
	SymbolSub	0.031	0.005	5.689	**1.29e-08**	**7.73e-08**	0.066
	DigitSpan	0.051	0.010	5.264	**1.43e-07**	**7.56e-07**	0.097
	TrailA	0.046	0.006	7.166	**7.93e-13**	**8.06e-12**	0.090
	TrailB	0.044	0.006	7.247	**4.37e-13**	**5.24e-12**	0.091
Miscarriage & Termination	PairsMatch	-0.020	0.014	-1.411	0.158	0.299	-0.020
	ReactionTime	0.012	0.013	0.888	0.375	0.515	0.012
	SymbolSub	0.015	0.021	0.707	0.480	0.597	0.017
	DigitSpan	0.028	0.043	0.639	0.523	0.616	0.029
	TrailA	0.014	0.023	0.594	0.552	0.634	0.015
	TrailB	-0.015	0.023	-0.652	0.515	0.612	-0.017
Stillbirth	PairsMatch	-0.001	0.080	-0.018	0.986	0.986	-0.001
	ReactionTime	0.037	0.075	0.494	0.621	0.683	0.040
	SymbolSub	-0.004	0.135	-0.030	0.976	0.984	-0.005
	DigitSpan	-0.169	0.259	-0.654	0.513	0.612	-0.176
	TrailA	-0.274	0.157	-1.747	0.081	0.172	-0.296
	TrailB	-0.280	0.152	-1.839	0.066	0.143	-0.312
Current HT use	PairsMatch	-0.031	0.011	-2.861	**0.004**	**0.014**	-0.026
	ReactionTime	0.012	0.010	1.126	0.260	0.404	0.010
	SymbolSub	-0.010	0.018	-0.555	0.579	0.659	-0.009
	DigitSpan	0.032	0.035	0.927	0.354	0.503	0.016

	TrailA	0.041	0.021	2.008	**0.045**	0.102	0.023
	TrailB	-0.025	0.020	-1.288	0.198	0.339	-0.015
Past HT use	PairsMatch	-0.033	0.006	-5.152	**2.58e-07**	**1.26e-06**	-0.047
	ReactionTime	0.025	0.006	4.170	**3.05e-05**	**1.30e-04**	0.039
	SymbolSub	-0.030	0.011	-2.777	**0.005**	**0.018**	-0.047
	DigitSpan	0.007	0.020	0.353	0.724	0.771	0.006
	TrailA	0.008	0.013	0.615	0.538	0.623	0.007
	TrailB	-0.053	0.012	-4.324	**1.53e-05**	**6.75e-05**	-0.049
Age HT initiation	PairsMatch	-0.003	0.005	-0.737	0.461	0.591	-0.006
	ReactionTime	0.005	0.005	1.188	0.235	0.378	0.010
	SymbolSub	0.019	0.008	2.365	**0.018**	**0.045**	0.040
	DigitSpan	0.015	0.014	1.041	0.298	0.442	0.028
	TrailA	0.001	0.010	0.095	0.924	0.952	0.002
	TrailB	0.007	0.010	0.765	0.444	0.575	0.014
Age HT rel. Menopause	PairsMatch	-0.004	0.005	-0.816	0.414	0.547	-0.008
	ReactionTime	0.001	0.005	0.187	0.852	0.892	0.002
	SymbolSub	0.010	0.010	1.081	0.280	0.425	0.021
	DigitSpan	-0.007	0.017	-0.427	0.669	0.724	-0.014
	TrailA	0.011	0.012	0.965	0.335	0.485	0.021
	TrailB	0.030	0.011	2.574	**0.010**	**0.029**	0.055
Duration HT use	PairsMatch	-0.004	0.005	-0.884	0.377	0.515	-0.008
	ReactionTime	0.004	0.005	0.981	0.326	0.479	0.009
	SymbolSub	-0.013	0.009	-1.505	0.132	0.264	-0.025
	DigitSpan	0.009	0.014	0.666	0.506	0.612	0.018
	TrailA	0.013	0.010	1.280	0.201	0.339	0.023
	TrailB	-0.013	0.010	-1.383	0.167	0.306	-0.025
Current HC use	PairsMatch	0.050	0.018	2.692	**0.007**	**0.022**	0.022
	ReactionTime	0.135	0.018	7.696	**1.41e-14**	**3.10e-13**	0.055
	SymbolSub	0.151	0.029	5.161	**2.46e-07**	**1.25e-06**	0.068
	DigitSpan	0.203	0.056	3.645	**2.68e-04**	**0.001**	0.074
	TrailA	0.162	0.033	4.866	**1.15e-06**	**5.21e-06**	0.070
	TrailB	0.195	0.032	6.134	**8.64e-10**	**6.34e-09**	0.089
Past HC use	PairsMatch	0.018	0.007	2.650	**0.008**	**0.024**	0.022
	ReactionTime	0.092	0.006	14.549	**6.34e-48**	**8.37e-46**	0.114
	SymbolSub	0.103	0.012	8.586	**9.30e-18**	**3.07e-16**	0.112
	DigitSpan	0.119	0.021	5.695	**1.26e-08**	**7.73e-08**	0.116
	TrailA	0.087	0.014	6.159	**7.40e-10**	**5.75e-09**	0.083
	TrailB	0.103	0.014	7.591	**3.25e-14**	**5.36e-13**	0.111
Age HC initiation	PairsMatch	-0.016	0.003	-5.113	**3.17e-07**	**1.50e-06**	-0.028
	ReactionTime	-0.028	0.003	-9.809	**1.04e-22**	**4.59e-21**	-0.053
	SymbolSub	-0.050	0.005	-9.878	**5.52e-23**	**3.64e-21**	-0.100
	DigitSpan	-0.071	0.010	-7.396	**1.48e-13**	**1.96e-12**	-0.125
	TrailA	-0.032	0.006	-5.334	**9.68e-08**	**5.33e-07**	-0.058

	TrailB	-0.043	0.006	-7.603	**2.98e-14**	**5.36e-13**	-0.082
Duration HC use	PairsMatch	0.004	0.003	1.361	0.173	0.314	0.008
	ReactionTime	0.008	0.003	2.826	**0.005**	**0.016**	0.016
	SymbolSub	0.036	0.005	7.877	**3.45e-15**	**9.11e-14**	0.082
	DigitSpan	0.049	0.009	5.670	**1.46e-08**	**8.39e-08**	0.099
	TrailA	0.035	0.005	6.745	**1.55e-11**	**1.37e-10**	0.075
	TrailB	0.029	0.005	5.745	**9.25e-09**	**6.43e-08**	0.064

Abbreviation: APOE = Apolipoprotein, PairMatch = Pair Matching Test, ReactionTime = Reaction Time Test, SymbolSub = Symbol Substitution Test, DigitSpan = Digit Span Test, Trail A & B = Trail Making Test A & B, N = Number, HT = Hormone Therapy, HC = Hormonal Contraceptive, S.E. = Standard Error, FDR = False Discovery Rate. Significant results are highlighted in bold.

Table S12| Associations between proxies of estrogen exposure and late life cognition after exclusion of participants with diagnosed brain diseases, autoimmune diseases, metabolic disorders and cancer.

Model	Test	beta	S.E.	t	p	p_{FDR}	d
Current HT use	PairsMatch	-0.031	0.012	-2.559	**0.010**	**0.022**	-0.026
	ReactionTime	0.008	0.012	0.702	0.483	0.606	0.007
	SymbolSub	-0.026	0.020	-1.297	0.195	0.304	-0.024
	DigitSpan	0.050	0.039	1.289	0.197	0.304	0.025
	TrailA	0.025	0.023	1.101	0.271	0.366	0.014
	TrailB	-0.028	0.022	-1.300	0.194	0.304	-0.017
Past HT use	PairsMatch	-0.032	0.007	-4.369	**1.25e-05**	**3.38e-05**	-0.045
	ReactionTime	0.030	0.007	4.382	**1.17e-05**	**3.34e-05**	0.047
	SymbolSub	-0.033	0.012	-2.715	**0.007**	**0.015**	-0.051
	DigitSpan	0.009	0.023	0.375	0.707	0.749	0.007
	TrailA	0.008	0.014	0.530	0.596	0.715	0.007
	TrailB	-0.054	0.014	-3.958	**7.56e-05**	**1.86e-04**	-0.050
Age HT initiation	PairsMatch	-0.001	0.005	-0.236	0.813	0.845	-0.002
	ReactionTime	-4.25e-05	0.005	-0.008	0.994	0.994	-8.05e-05
	SymbolSub	0.014	0.009	1.537	0.124	0.224	0.029
	DigitSpan	0.021	0.017	1.275	0.202	0.304	0.040
	TrailA	-0.005	0.011	-0.426	0.670	0.747	-0.009
	TrailB	0.005	0.011	0.454	0.650	0.747	0.009
Age HT rel. Menopause	PairsMatch	-0.002	0.006	-0.409	0.682	0.747	-0.005
	ReactionTime	0.000	0.006	-0.046	0.963	0.981	-0.001
	SymbolSub	0.014	0.011	1.243	0.214	0.304	0.028
	DigitSpan	-0.011	0.019	-0.542	0.588	0.715	-0.020
	TrailA	0.017	0.013	1.249	0.212	0.304	0.030
	TrailB	0.029	0.013	2.217	**0.027**	*0.050*	0.054
Duration HT use	PairsMatch	-0.005	0.005	-0.925	0.355	0.467	-0.009
	ReactionTime	0.007	0.005	1.401	0.161	0.280	0.014
	SymbolSub	-0.014	0.010	-1.385	0.166	0.280	-0.026
	DigitSpan	0.006	0.016	0.396	0.692	0.747	0.013
	TrailA	0.010	0.011	0.901	0.367	0.472	0.018
	TrailB	-0.012	0.011	-1.110	0.267	0.366	-0.023
Current HC use	PairsMatch	0.044	0.019	2.263	**0.024**	**0.046**	0.019
	ReactionTime	0.149	0.018	8.096	**5.72e-16**	**7.73e-15**	0.061
	SymbolSub	0.173	0.031	5.607	**2.07e-08**	**7.98e-08**	0.076
	DigitSpan	0.194	0.058	3.336	**0.001**	**0.002**	0.077
	TrailA	0.155	0.035	4.421	**9.85e-06**	**2.96e-05**	0.070
	TrailB	0.221	0.033	6.611	**3.87e-11**	**2.32e-10**	0.106
Past HC use	PairsMatch	0.019	0.008	2.570	**0.010**	**0.022**	0.021
	ReactionTime	0.094	0.007	13.106	**3.24e-39**	**1.75e-37**	0.104
	SymbolSub	0.103	0.013	7.756	**8.96e-15**	**8.07e-14**	0.106

	DigitSpan	0.124	0.024	5.199	**2.04e-07**	**7.33e-07**	0.120
	TrailA	0.094	0.016	6.014	**1.83e-09**	**8.25e-09**	0.091
	TrailB	0.117	0.015	7.791	**6.85e-15**	**7.40e-14**	0.125
Age HC initiation	PairsMatch	-0.014	0.003	-4.158	**3.21e-05**	**8.25e-05**	-0.025
	ReactionTime	-0.029	0.003	-9.178	**4.45e-20**	**1.20e-18**	-0.055
	SymbolSub	-0.044	0.005	-8.135	**4.25e-16**	**7.65e-15**	-0.089
	DigitSpan	-0.061	0.010	-5.915	**3.42e-09**	**1.42e-08**	-0.110
	TrailA	-0.031	0.006	-4.902	**9.54e-07**	**3.03e-06**	-0.057
	TrailB	-0.041	0.006	-6.698	**2.16e-11**	**1.46e-10**	-0.078
Duration HC use	PairsMatch	0.001	0.003	0.397	0.692	0.747	0.002
	ReactionTime	0.007	0.003	2.502	**0.012**	**0.025**	0.015
	SymbolSub	0.036	0.005	7.235	**4.75e-13**	**3.67e-12**	0.081
	DigitSpan	0.049	0.009	5.166	**2.43e-07**	**8.21e-07**	0.099
	TrailA	0.036	0.006	6.314	**2.76e-10**	**1.49e-09**	0.076
	TrailB	0.033	0.005	6.074	**1.26e-09**	**6.21e-09**	0.073

Abbreviation: PairMatch = Pair Matching Test, ReactionTime = Reaction Time Test, SymbolSub = Symbol Substitution Test, DigitSpan = Digit Span Test, Trail A & B = Trail Making Test A & B, N = Number, HT = Hormone Therapy, HC = Hormonal Contraceptive, S.E. = Standard Error, FDR = False Discovery Rate. Significant results are highlighted in bold.

Table S13| Detected extreme values of continuous female-specific factors using the median absolute deviation method.

				Number extreme values		
**Variable**	**Median**	**MAD**	**Limits***	**Low**	**High**	**Total**
Age at Menarche	13	1.48	8.55 – 17.45	130	920	1050
Age at Menopause	50	4.45	36.66 – 63.34	2200	24	2224
Age at Bilateral Oophorectomy	48	7.41	25.76 – 70.24	51	0	51
Age at Hysterectomy	44	7.41	21.76 – 66.24	26	140	166
Number of live Childbirths	2	1.48	-2.45 – 6.45	0	322	322
Age at first Childbirth	25	4.45	11.66 – 38.34	1	790	791
Age at last Childbirth	30	4.45	16.66 – 43.34	7	497	504
Age started HT	48	4.45	34.66 – 61.34	1185	115	1300
Age last used HT	55	5.93	37.21 – 72.79	537	0	537
Age started HC	21	4.45	7.66 – 34.34	10	3364	3374
Age last used HC	31	7.41	8.76 – 53.24	2	741	743

*Limits of acceptable range of values. Abbreviations: HT = hormone therapy, HC = hormonal contraceptives, MAD = median absolute deviation.

Table S14| Main effects of APOE ε4 genotype on cognitive performance.

**Model**	**Test**	**beta**	**S.E.**	**t**	**p**	**pFDR**	**d**
APOE ε4	PairsMatch	-0.010	0.005	-1.873	0.061	0.123	-0.010
	ReactionTime	-0.001	0.005	-0.162	0.871	0.925	-0.001
	SymbolSub	-0.046	0.009	-4.974	**6.59e-07**	**5.93e-06**	-0.053
	DigitSpan	-0.031	0.017	-1.823	0.068	0.123	-0.032
	TrailA	-0.014	0.011	-1.267	0.205	0.284	-0.014
	TrailB	-0.027	0.010	-2.673	**0.008**	**0.027**	-0.030
1x ε4 allele	PairsMatch	-0.008	0.006	-1.417	0.156	0.242	-0.008
2x ε4 allele	PairsMatch	-0.033	0.016	-2.060	**0.039**	0.118	-0.012
1x ε4 allele	ReactionTime	-0.001	0.005	-0.145	0.884	0.925	-0.001
2x ε4 allele	ReactionTime	-0.001	0.015	-0.094	0.925	0.925	-0.001
1x ε4 allele	SymbolSub	-0.037	0.010	-3.840	**1.23e-04**	**0.001**	-0.042
2x ε4 allele	SymbolSub	-0.145	0.027	-5.276	**1.33e-07**	**2.39e-06**	-0.057
1x ε4 allele	DigitSpan	-0.034	0.018	-1.964	0.049	0.123	-0.086
2x ε4 allele	DigitSpan	0.005	0.049	0.109	0.913	0.925	0.005
1x ε4 allele	TrailA	-0.011	0.011	-0.962	0.336	0.432	-0.028
2x ε4 allele	TrailA	-0.045	0.032	-1.401	0.161	0.242	-0.040
1x ε4 allele	TrailB	-0.020	0.011	-1.852	0.064	0.123	-0.055
2x ε4 allele	TrailB	-0.111	0.031	-3.619	**2.96e-04**	**0.001**	-0.107

Abbreviation: APOE = Apolipoprotein, PairMatch = Pair Matching Test, ReactionTime = Reaction Time Test, SymbolSub = Symbol Substitution Test, DigitSpan = Digit Span Test, Trail A & B = Trail Making Test A & B, N = Number, HT = Hormone Therapy, HC = Hormonal Contraceptive, S.E. = Standard Error, FDR = False Discovery Rate. Significant results are highlighted in bold.

Table 15| Interactions between APOE ε4 genotype and female-specific factors on late life cognition.

**Model**	**Test**	**beta**	**S.E.**	**t**	**p**	**p_{FDR}**
Reproductive Span * *APOE* ε4	PairsMatch	-0.002	0.002	-0.783	0.434	0.867
	ReactionTime	0.001	0.002	0.494	0.621	0.867
	SymbolSub	0.006	0.004	1.353	0.176	0.867
	DigitSpan	0.007	0.007	0.987	0.324	0.867
	TrailA	-0.007	0.005	-1.359	0.174	0.867
	TrailB	0.003	0.005	0.671	0.502	0.867
Age at Menarche * *APOE* ε4	PairsMatch	0.003	0.007	0.514	0.607	0.867
	ReactionTime	-0.005	0.006	-0.752	0.452	0.867
	SymbolSub	-0.001	0.012	-0.047	0.963	0.983
	DigitSpan	0.008	0.020	0.394	0.693	0.897
	TrailA	0.003	0.014	0.218	0.828	0.966
	TrailB	0.007	0.013	0.499	0.617	0.867
Age at Menopause * *APOE* ε4	PairsMatch	-0.004	0.007	-0.598	0.550	0.867
	ReactionTime	0.004	0.006	0.569	0.569	0.867
	SymbolSub	-0.008	0.012	-0.706	0.480	0.867
	DigitSpan	0.019	0.021	0.929	0.353	0.867
	TrailA	-0.002	0.014	-0.160	0.873	0.968
	TrailB	0.011	0.013	0.826	0.409	0.867
Oophorectomy * *APOE* ε4	PairsMatch	-0.002	0.014	-0.119	0.906	0.977
	ReactionTime	-0.029	0.013	-2.210	0.027	0.596
	SymbolSub	0.023	0.021	1.098	0.272	0.867
	DigitSpan	-0.028	0.043	-0.653	0.514	0.867
	TrailA	0.031	0.024	1.322	0.186	0.867
	TrailB	0.013	0.023	0.553	0.580	0.867
Age at Oophorectomy * *APOE* ε4	PairsMatch	-0.003	0.003	-1.033	0.301	0.867
	ReactionTime	0.005	0.003	1.997	0.046	0.755
	SymbolSub	-0.001	0.005	-0.220	0.826	0.966
	DigitSpan	-0.008	0.008	-0.974	0.330	0.867
	TrailA	0.004	0.006	0.798	0.425	0.867
	TrailB	0.004	0.005	0.821	0.411	0.867
Hysterectomy * *APOE* ε4	PairsMatch	0.090	0.082	1.097	0.273	0.867
	ReactionTime	-0.125	0.078	-1.609	0.108	0.867
	SymbolSub	0.127	0.170	0.743	0.457	0.867
	DigitSpan	-0.400	0.231	-1.729	0.084	0.867
	TrailA	0.335	0.183	1.829	0.067	0.862
	TrailB	0.058	0.178	0.324	0.746	0.947
Age at Hysterectomy * *APOE* ε4	PairsMatch	0.002	0.003	0.697	0.486	0.867
	ReactionTime	-0.006	0.003	-2.245	0.025	0.596
	SymbolSub	-0.003	0.004	-0.605	0.545	0.867
	DigitSpan	0.006	0.008	0.674	0.501	0.867

	TrailA	-0.009	0.005	-1.800	0.072	0.862
	TrailB	-0.001	0.005	-0.115	0.908	0.977
N Childbirth * *APOE* ε4	PairsMatch	-0.008	0.011	-0.718	0.473	0.867
	ReactionTime	0.039	0.011	3.589	**3.32e-04**	**0.022**
	SymbolSub	0.031	0.022	1.436	0.151	0.867
	DigitSpan	0.012	0.041	0.294	0.769	0.958
	TrailA	-0.022	0.025	-0.886	0.375	0.867
	TrailB	-0.021	0.024	-0.887	0.375	0.867
N Childbirth^2 * *APOE* ε4	PairsMatch	0.009	0.012	0.721	0.471	0.867
	ReactionTime	-0.046	0.012	-3.831	**1.28e-04**	**0.017**
	SymbolSub	-0.032	0.027	-1.182	0.237	0.867
	DigitSpan	-0.040	0.047	-0.838	0.402	0.867
	TrailA	0.015	0.031	0.477	0.633	0.867
	TrailB	0.001	0.030	0.047	0.963	0.983
Age last Birth * *APOE* ε4	PairsMatch	0.043	0.021	2.054	0.040	0.755
	ReactionTime	0.000	0.020	-0.002	0.999	0.999
	SymbolSub	0.027	0.038	0.716	0.474	0.867
	DigitSpan	0.037	0.065	0.567	0.571	0.867
	TrailA	-0.002	0.045	-0.045	0.964	0.983
	TrailB	0.042	0.043	0.962	0.336	0.867
Age first Birth * *APOE* ε4	PairsMatch	-0.005	0.021	-0.234	0.815	0.966
	ReactionTime	0.013	0.020	0.656	0.512	0.867
	SymbolSub	-0.007	0.039	-0.179	0.858	0.966
	DigitSpan	-0.068	0.061	-1.107	0.269	0.867
	TrailA	0.004	0.047	0.092	0.927	0.983
	TrailB	0.009	0.046	0.205	0.837	0.966
Miscarriage & Termination * *APOE* ε4	PairsMatch	-0.025	0.019	-1.271	0.204	0.867
	ReactionTime	0.003	0.018	0.149	0.881	0.970
	SymbolSub	-0.030	0.034	-0.859	0.390	0.867
	DigitSpan	0.055	0.062	0.887	0.375	0.867
	TrailA	-0.015	0.041	-0.374	0.709	0.908
	TrailB	-0.016	0.039	-0.414	0.679	0.896
Stillbirth * *APOE* ε4	PairsMatch	0.020	0.019	1.062	0.288	0.867
	ReactionTime	0.011	0.018	0.600	0.549	0.867
	SymbolSub	0.021	0.034	0.618	0.536	0.867
	DigitSpan	0.038	0.062	0.608	0.543	0.867
	TrailA	-0.033	0.042	-0.783	0.434	0.867
	TrailB	-0.002	0.040	-0.061	0.951	0.983
Current HT use * *APOE* ε4	PairsMatch	-0.007	0.010	-0.627	0.531	0.867
	ReactionTime	0.007	0.010	0.741	0.459	0.867
	SymbolSub	-0.018	0.017	-1.067	0.286	0.867
	DigitSpan	-0.025	0.034	-0.737	0.461	0.867
	TrailA	-0.014	0.020	-0.711	0.477	0.867

	TrailB	-0.010	0.019	-0.530	0.596	0.867
Past HT use * *APOE* ε4	PairsMatch	0.003	0.006	0.459	0.646	0.867
	ReactionTime	0.008	0.005	1.486	0.137	0.867
	SymbolSub	-0.022	0.010	-2.275	0.023	0.596
	DigitSpan	-0.009	0.017	-0.539	0.590	0.867
	TrailA	0.003	0.011	0.236	0.814	0.966
	TrailB	-0.001	0.011	-0.113	0.910	0.977
Age HT initiation * *APOE* ε4	PairsMatch	-0.004	0.004	-0.908	0.364	0.867
	ReactionTime	0.003	0.004	0.712	0.476	0.867
	SymbolSub	-0.012	0.008	-1.518	0.129	0.867
	DigitSpan	-0.003	0.013	-0.239	0.811	0.966
	TrailA	-0.006	0.010	-0.634	0.526	0.867
	TrailB	-0.002	0.009	-0.248	0.804	0.966
Age HT rel. Menopause * *APOE* ε4	PairsMatch	-0.002	0.004	-0.458	0.647	0.867
	ReactionTime	0.002	0.004	0.571	0.568	0.867
	SymbolSub	-0.004	0.008	-0.467	0.640	0.867
	DigitSpan	-0.003	0.013	-0.198	0.843	0.966
	TrailA	0.007	0.009	0.809	0.419	0.867
	TrailB	0.000	0.009	-0.031	0.975	0.983
Duration HT use * *APOE* ε4	PairsMatch	-0.005	0.005	-1.067	0.286	0.867
	ReactionTime	0.004	0.005	0.796	0.426	0.867
	SymbolSub	0.000	0.009	-0.032	0.975	0.983
	DigitSpan	-0.003	0.016	-0.172	0.864	0.966
	TrailA	0.019	0.012	1.645	0.100	0.867
	TrailB	0.022	0.011	1.944	0.052	0.761
Current HC use * *APOE* ε4	PairsMatch	0.005	0.018	0.306	0.759	0.955
	ReactionTime	-0.008	0.017	-0.454	0.650	0.867
	SymbolSub	0.066	0.028	2.354	0.019	0.596
	DigitSpan	0.064	0.054	1.178	0.239	0.867
	TrailA	0.001	0.032	0.041	0.968	0.983
	TrailB	0.048	0.031	1.546	0.122	0.867
Past HC use * *APOE* ε4	PairsMatch	0.003	0.006	0.479	0.632	0.867
	ReactionTime	-0.001	0.006	-0.180	0.857	0.966
	SymbolSub	0.017	0.012	1.418	0.156	0.867
	DigitSpan	0.033	0.021	1.573	0.116	0.867
	TrailA	0.019	0.014	1.346	0.178	0.867
	TrailB	0.018	0.013	1.305	0.192	0.867
Age HC initiation * *APOE* ε4	PairsMatch	0.003	0.003	1.056	0.291	0.867
	ReactionTime	-0.001	0.003	-0.218	0.827	0.966
	SymbolSub	-0.003	0.006	-0.518	0.605	0.867
	DigitSpan	0.008	0.010	0.791	0.429	0.867
	TrailA	-0.006	0.007	-0.799	0.424	0.867
	TrailB	-0.006	0.007	-0.804	0.421	0.867

Duration HC use * *APOE* ε4	PairsMatch	0.004	0.003	1.186	0.236	0.867
	ReactionTime	-0.002	0.003	-0.755	0.450	0.867
	SymbolSub	-0.004	0.006	-0.667	0.505	0.867
	DigitSpan	0.004	0.010	0.398	0.691	0.897
	TrailA	-0.004	0.007	-0.504	0.614	0.867
	TrailB	-0.004	0.007	-0.507	0.612	0.867

Abbreviation: APOE = Apolipoprotein. PairMatch = Pair Matching Test. ReactionTime = Reaction Time Test. SymbolSub = Symbol Substitution Test. DigitSpan = Digit Span Test. Trail A & B = Trail Making Test A & B. N = Number. HT = Hormone Therapy. HC = Hormonal Contraceptive. S.E. = Standard Error. FDR = False Discovery Rate. Significant results are highlighted in bold.

1. Foster HME, Celis-Morales CA, Nicholl BI, Petermann-Rocha F, Pell JP, Gill JMR *et al.* The effect of socioeconomic deprivation on the association between an extended measurement of unhealthy lifestyle factors and health outcomes: a prospective analysis of the UK Biobank cohort. *Lancet Public Health* 2018; **3**(12)**:** e576-e585.
